# Tunable quantum light by modulated free electrons

**DOI:** 10.1515/nanoph-2025-0040

**Published:** 2025-05-22

**Authors:** Valerio Di Giulio, Rudolf Haindl, Claus Ropers

**Affiliations:** Department of Ultrafast Dynamics, Max Planck Institute for Multidisciplinary Sciences, D-37077 Göttingen, Germany; 4th Physical Institute – Solids and Nanostructures, Georg-August-Universität Göttingen, D-37077 Göttingen, Germany

**Keywords:** free electrons, quantum optics, ultrafast, electron microscopy, photonics

## Abstract

Nonclassical states of light are fundamental in various applications, spanning quantum computation to enhanced sensing. Fast free electrons, which emit light into photonic structures through the mechanism of spontaneous emission, represent a promising platform for generating diverse types of states. Indeed, the intrinsic connection between the input electron wave function and the output light field suggests that electron-shaping schemes, based on light-induced scattering, facilitate their synthesis. In this article, we present a theoretical framework capable of predicting the final optical density matrix emitted by a generic *N*-electron state that can also account for post-sample energy filtering. By using such a framework, we study the modulation-dependent fluctuations of the *N*-electron emission and identify regions of superradiant scaling characterized by Poissonian and super-Poissonian statistics. In this context, we predict that high-*N* modulated electron pulses can yield a tenfold shot-noise suppression in the estimation of the electron-light coupling when the output radiation intensity is analyzed. In the single-electron case, we show how coherent states with nearly 90 % purity can be formed by pre-filtering a portion of the spectrum after modulation, and how non-Gaussian states are generated after a precise energy measurement. Furthermore, we present a strategy combining a single-stage electron modulation and post-filtering to harness tailored light states, such as squeezed vacuum, cat, and triangular cat states, with fidelities close to 100 %.

## 1 Introduction

Fast electrons in scanning and transmission electron microscopes (SEM/TEM) offer the capability to measure different material properties with nanometer resolution, thanks to their exceptionally small wavelength. For instance, inelastically scattered electrons carry information about the excitations of a sample, such as phonons [1], [2], plasmonic resonances [3], [4], [5], [6], and geometrically confined dielectric modes [7], [8], which can be retrieved by analyzing their final spectrum through electron energy-loss spectroscopy (EELS) [9], [10].

In the past two decades, efforts to improve the spectral resolution, limited in EELS measurements by the broad-band nature of fast charged particles [9], and to achieve time-resolved imaging, have led to the integration of optical systems into TEM. In such instruments, a laser and an electron pulse interact at the sample, resulting in inelastic electron-light scattering (IELS) [11], [12]. In the form of photon-induced near-field electron microscopy (PINEM), this combination of techniques has produced remarkable results in studying the femtosecond dynamics of near fields carried by polaritons in nanostructures [13], [14], [15], [16], [17] and optical nonlinearities in dielectric resonators [18]. Beyond imaging, IELS has proven to be an important phenomenon for coherently shaping the longitudinal [19] and transverse [20], [21] full three-dimensional wave function of an electron beam (e-beam). In this context, a general IELS interaction with laser frequency *ω*_L_ near a plane positioned at *z* along the propagation axis, brings an electron traveling with velocity *v* into the superposition state

$$\psi_{e}\left(z\right)=\psi_{0}\left(z\right)\;\;\overset{\infty }{\underset{\ell =-\infty }{\sum }}\;\;c_{\ell }\;e^{i\ell \omega_{\text{L}}z/v}$$

composed of energy coefficients *c*_*ℓ*_ and an envelope *ψ*_0_(*z*). Controlling the amplitude and phase of these coefficients is crucial for attosecond bunching of the electron density [22], [23], [24]. Several schemes combining multiple IELS interaction zones have been proposed [16], [25], [26], [27], [28] to achieve extreme temporal compression, including the replacement of laser illumination with a quantum light source [29], [30], [31].

Free electrons in SEM/TEM also represent a unique platform for tailoring and probing quantum characteristics of polaritonic modes, either confined, or guided within photonic structures [32], [33], [34], [35]. In the case of bosonic statistics, it was shown that the incoming electron energy coefficients *c*_*ℓ*_ and the output mode density matrix *ρ*_*p*_ are directly related [30], [36], thus rendering a tailored IELS modulation an excellent means to control the latter. Under the usual conditions of electron-light coupling linear in the electric field of the mode [29], [32], Poissonian-distributed emission is predicted to arise from single-electron pulses, with a state purity determined by the temporal structure of the electron density [30]. Since a possible way of generating quantum light exploits a nonlinear interaction, schemes based on quadratic ponderomotive coupling to produce squeezing [37] or incorporating final electron energy filtering (post-filtering) have been proposed [36], [38] and applied to herald few-photon Fock states [39], [40]. Furthermore, more complex light states, such as cat and GKP states [41], were shown to be producible by employing multiple electrons shaped into idealized electron superpositions, characterized by energy coefficients with constant amplitudes at all orders and with corresponding phases ∝*ℓ* [42].

This article aims at exploring in detail the connection between electron energy modulation and light emission in a single photonic mode with a particular focus on quantum light synthesis. The work is organized as follows. In Section 2.1, we develop a general theoretical framework for a linear type – with an interaction Hamiltonian proportional to the mode electric field – of electron-light coupling capable of connecting, through an input-output relation, an incoming *N*-electron density matrix with *ρ*_*p*_. In addition, the action of an electron spectrometer is incorporated in the theory to account for the possibility of energy post-filtering. Without post-filtering, we predict super-Poissonian light emission arising from *N* > 1 bunches for most electron modulations and Poissonian statistics in specific limiting cases. We then apply parameter estimation theory to study how these types of electron pulses affect shot-noise limited measurements of electron-light interaction strengths. In Section 2.2, we analyze, for single-electron pulses, the coherence conditions and the corresponding modulation requirements to generate high-purity states, both with and without post-filtering. By focusing on the latter case, we propose a simple modulation scheme that combines a strong IELS interaction with an energy filter placed before the sample to significantly enhance electron coherence and state purity. Moreover, for electrons with coherence times longer than the optical cycle of the mode and incorporating post-filtering, we show that pure light states are produced regardless of the form of *c*_*ℓ*_. In Section 2.3, we leverage the implications of the previous result to explore how a standard IELS modulation can create cat states. Subsequently, in Section 2.4, we adopt an approach used for electron-pulse shaping [26] combined with an optimization algorithm to provide specific guidelines for designing near-field distributions to be used in an IELS interaction leading to the synthesis of more complex light states. We find that squeezed vacuum, cat, and triangular cat states can be generated with ∼100 % fidelity under strong coupling conditions and with modulation parameters accessible to state-of-the-art setups. Finally in Section 3, we discuss the results, their possible extensions, and we provide considerations on the application of the proposed strategies.

In addition to their theoretical significance, our results represent a fundamental step towards developing practical methods for harnessing nonclassical light from free electrons.

## 2 Results and discussion

### 2.1 Output light density matrix after interaction with *N* electrons

**Figure 1:**
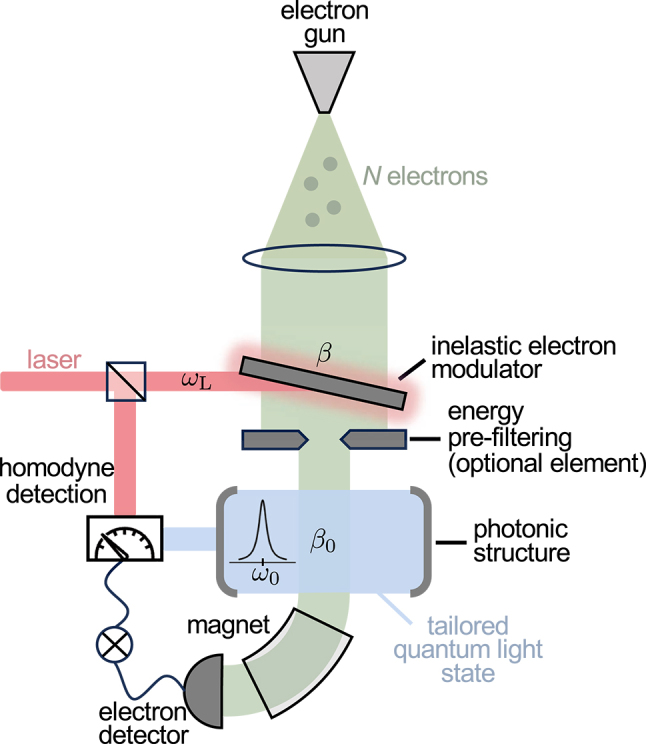
Creation and analysis of quantum light states generated by free electrons. An e-beam pulse composed by *N* electrons is directed into a light-based inelastic modulator that coherently reshapes the electron energy distribution through a single IELS interaction with coupling coefficient *β* and frequency *ω*_L_. An optional energy filter placed before the sample may eliminate electrons outside a selected energy range. The beam subsequently passes a nanostructure and emits photons into an optical mode with frequency *ω*_0_ via spontaneous emission of strength *β*_0_. After this interaction, the generated light is extracted from the structure, and its quantum state is analyzed using a homodyne detection scheme in coincidence with the energies measured by an electron spectrometer composed by a sector magnet and an electron detector.

In this work, we study the quantum properties of light emitted in a photonic structure by the interaction of an e-beam at kinetic energies in the keV range with a single optical mode of energy *ℏω*_0_ and an electric field profile ${\vec{E}}_{0}\left(r\right)$ . In particular, we are interested in computing the post-interaction light density matrix *ρ*_*p*_ for electrons having passed through a modulation stage that may comprise an IELS interaction and an energy filter before the sample (pre-filtering). Moreover, we consider the consequences linked to light generation when only a subset of events, determined by a particular choice of the electrons’ final energies, is considered (post-filtering) (see Figure 1). In doing so, we will assume each e-beam pulse to contain *N* electrons, all with central velocity $\text{v}=v\hat{z}$ corresponding to a kinetic energy $E_{0}^{e}\gg \hbar \omega_{0}$ , and to be well-focused around the transversal coordinate **R**.

Under these conditions, the quantum evolution of the joint electron-light state can be written by linearizing the electron dispersion, directly leading to the closed form of the scattering operator $\hat{S}=e^{i\hat{\chi }}\;\hat{U}$ (see Supplementary Information (SI) [43]), with

(1) $$\hat{U}=e^{\beta_{0}\left(\hat{b}{\hat{a}}^{†}-{\hat{b}}^{†}\hat{a}\right)},$$

written in terms of the electron ${\hat{b}}^{†}$ , $\hat{b}$ and the photon ${\hat{a}}^{†},\hat{a}$ creation and annihilation operators. While $\hat{a},{\hat{a}}^{†}$ act on the number of photons, subtracting and adding one particle, respectively, $\hat{b}$ decreases and ${\hat{b}}^{†}$ increases the longitudinal momentum of one of the electrons in the bunch by *ω*_0_/*v*, i.e., bringing any *N*-electron momentum eigenstate |*q*_1_, …, *q*_*N*_⟩ to the superposition $\sum_{i=1}^{N}|q_{1},\ldots ,q_{i}\pm \omega_{0}/v,\ldots ,q_{N}〉$ . In particular, the former follow boson statistics, whereas, in the considered nonrecoil approximation, which is well justified at high electron energies, the latter commute $\left[\hat{b},{\hat{b}}^{†}\right]=0$ [19], [29], [44]. The coupling coefficient $\beta_{0}=\left(e/\hbar \omega_{0}\right)\left|\int_{-\infty }^{\infty }dz\;E_{0,z}\left(R,z\right)\;e^{-i\omega_{0}z/v}\right|$ (sometimes referred to as *g*_*Q*_ in the literature [29], [36], [39]) determines the number of photons exchanged between the electron and the optical mode and can be evaluated through standard methods employed to compute EELS probabilities [45]. We remark that $\hat{S}$ connects the density matrix prior to the scattering in the interaction picture *ρ*(−∞) with the state after the interaction *ρ*(∞) as $\rho \left(\infty \right)=\hat{S}\rho \left(-\infty \right){\hat{S}}^{†}$ . The operator $\hat{\chi }$ accounts for the non-resonant part of the electron-electron interaction mediated by the surrounding dielectric environment and induces an elastic phase shift on the wave function of a single electron passing close to a conductive surface [46]. Owing to its short time scale in the few-fs range and the typical temporal separation between electrons of hundreds of fs, we disregard its effect in the rest of this work. Interestingly, the electron-light entanglement generated by the excitation-number-conserving evolution operator of Eq. (1) has recently been demonstrated in a TEM through a quantum eraser experiment [47].

The single-mode assumption, underlying the validity of Eq. (1), strongly depends on the value of *β*_0_ for the coupling to each mode allowed by the material and the configuration details of the photonic structure collecting the electron emission. Generally, narrow-band selectivity can be achieved in one-dimensional geometries through phase-matching, when the mode’s phase velocity *ω*_0_/*k*_0_ equals the electron group velocity *v*, i.e., when *ω*_0_/*k*_0_ ∼ *v* [39], [40], [48], [49]. However, somewhat weaker selectivity can also be achieved in confined resonances supported by nanostructures [50], [51].

To compute the statistical properties of the light emitted by electrons measured in a final set of longitudinal momenta **q**_*N*_ = (*q*_1_, …, *q*_*N*_), we begin with the calculation of the matrix $T^{q_{N}}=\left\langle q_{N}|\;\hat{U}\;\rho \left(-\infty \right)\;{\hat{U}}^{†}|q_{N}\right\rangle$ , which is a key intermediate in the derivation of the optical density matrix. Indeed, it projects the evolved quantum state of the system (after interaction) onto the electron momentum eigenstates. Interestingly, its evaluation becomes straightforward when performed in the spatial representation $|z_{N}〉=\sum_{q_{N}}\left(e^{-iq_{N}\cdot z_{N}}/L^{N/2}\right)|q_{N}〉$ (where *L* is the quantization length), as these states satisfy the eigenequations $\hat{b}|z_{N}〉=j\left(z_{N}\right)|z_{N}〉$ and ${\hat{b}}^{†}|z_{N}〉=j^{*}\left(z_{N}\right)|z_{N}〉$ with $j\left(z_{N}\right)=\sum_{i=1}^{N}e^{-i\omega_{0}z_{i}/v}$ . In physical terms, j*(**z**_*N*_) represents the *ω*_0_-frequency contribution of a classical current in units of −*e* formed by *N* electrons longitudinally distributed as the components of **z**_*N*_. As such, it is an eigenvalue of the current operator of negative frequency, which is proportional to ${\hat{b}}^{†}$ [44].

Under typical experimental conditions, the optical mode is either in the vacuum state or excited with a laser, while the *N*-electron bunch exists in a complex state arising from an incoherent ensemble average over stochastic fluctuations of the electron source, combined with the coherent operations of IELS modulation and energy pre-filtering. To best describe such initial conditions, we set as pre-interaction electron-light state $\left\langle z_{N}|\rho \left(-\infty \right)|z_{N}^{\prime }\right\rangle =\rho_{e}\left(z_{N},z_{N}^{\prime }\right)\;|\alpha 〉〈\alpha |$ , where |*α*⟩ is a bosonic coherent state of the mode with amplitude *α*, and $\rho_{e}\left(z_{N},z_{N}^{\prime }\right)$ is the spatial representation of the *N*-electron density matrix. Generally, electron sources triggered by photoemission pulses generate states populated by a fluctuating number of electrons, with a mean that is controlled by the incident laser. However, in this work, we restrict our analysis to the fixed number *N*, owing to the capabilities of cutting-edge experimental setups equipped with number-resolved electron detectors [40], [52], [53]. We remark that predictions involving a fluctuating number of electrons may be computed by averaging our results over the electron source distribution.

To account for general multi-electron post-filtering performed over a finite set of final momenta, we introduce the dimensionless detector function *F*(**q**_*N*_) which vanishes for values of **q**_*N*_ outside the selected region. By integrating the product $F\left(q_{N}\right)T^{q_{N}}$ , we can write the exact form of the output light density matrix after the interaction (see SI [43] for a detailed calculation):

(2) $$\begin{array}{ll} \rho_{p} & =\frac{1}{P_{F}}\int \;dz_{N}dz_{N}^{\prime }F\left(z_{N}-z_{N}^{\prime }\right)\rho_{e}\left(z_{N},z_{N}^{\prime }\right)\\ & \;\times \left|\alpha +\beta_{0}j\left(z_{N}\right)\right\rangle \left\langle \alpha +\beta_{0}j\left(z_{N}^{\prime }\right)\right.|, \end{array}$$

where the function $F\left(z_{N}\right)=\int dq_{N}F\left(q_{N}\right)\;e^{-iq_{N}\cdot z_{N}}/{\left(2\pi \right)}^{N}$ represents the detector response function. The normalization constant *P*_*F*_ ≤ 1 corresponds to the probability of success of the post-filtering operation as well as to the *N*-electron energy correlations developed during the light-mediated coupling [29], [54]. Importantly, Eq. (2) establishes a direct connection between a generic incoming *N*-electron state and the created light state. Interestingly, the final optical density matrix is formed by a continuous superposition of coherent states with amplitudes determined by classical multi-electron currents and coefficients determined by the incoming *N*-electron state and the detector response function. Furthermore, Eq. (2) highlights that a complete tomography of *ρ*_*p*_ could enable full readout of $\rho_{e}\left(z_{N},z_{N}^{\prime }\right)$ , including the retrieval of quantum entanglement between the momentum states of different electrons. An entanglement that has also been predicted to cause visible variations in the cathodoluminescence emission pattern when no post-filtering is applied [55].

Note that, if no post-filtering is performed $\left[F\left(z_{N}-z_{N}^{\prime }\right)=\delta \left(z_{N}-z_{N}^{\prime }\right)\right]$ , Eq. (2) shows that *ρ*_*p*_ becomes a function of the *N*-electron density *ρ*_*e*_(**z**_*N*_, **z**_*N*_) only. In this regime, the evaluation of expectation values of normally-ordered light operators is made particularly simple. For high electron currents, Coulomb interaction through the propagation in TEM can induce marked electron-electron transversal and longitudinal energy correlations, as shown by a recent experiment measuring the ensemble properties of few-electron bunches [52], [56]. While Eq. (2) maintains its validity under these conditions, for illustrative purposes and to derive example results, in the following we assume uncorrelated particles, which is the case when sufficiently spaced electrons in time arrive at the sample. In this scenario, the total density factorizes as $\rho_{e}\left(z_{N},z_{N}\right)=\prod_{i=1}^{N}\rho_{e}^{i}\left(z_{i},z_{i}\right)$ , and all light properties depend on the so-called electron coherence factor (CF) [57], [58].

(3) $$M_{k}^{i}=\overset{\infty }{\underset{-\infty }{\int }}\;\;\;dz\;\rho_{e}^{i}\left(z,z\right)\;e^{ikz}.$$

The CF is a measure of the coherence carried by each of the electrons at momentum *k*, quantified through the strength of the Fourier components of their densities. In practice, it defines the ability of the light emitted by the electrons to interfere with a second time-varying signal [44], [58]. For instance, if all electrons share the same density $\left(M_{k}^{i}\equiv M_{k}\right)$ , the total radiated intensity in the absence of laser excitation takes the form $I_{N}=\langle \hat{n}\rangle =\left\langle {\hat{a}}^{†}\hat{a}\right\rangle =\beta_{0}^{2}N\left[1+\left(N-1\right)|M_{\omega_{0}/v}{|}^{2}\right]$ and scales as *N* ^2^ when the CF approaches unity. This multi-electron cooperative effect, where the interfering fields are mutually generated by the electrons, produces an emission intensity ∝*N* ^2^, resembling the Schwartz–Hora effect [59], and is referred to as superradiance [60], [61]. Such behavior has been experimentally observed in transition radiation [62] and lies at the core of free-electron laser operation [63], [64], [65]. The type of emission is also characterized by its intensity fluctuations $\Delta I_{N}^{2}=\left\langle {\hat{n}}^{2}\right\rangle -I_{N}^{2}$ that read

(4) $$\Delta I_{N}^{2}/I_{N}=1+I_{N}\left[g^{\left(2\right)}\left(0\right)-1\right],$$

where $g^{\left(2\right)}\left(0\right)=\left\langle {\hat{a}}^{†2}{\hat{a}}^{2}\right\rangle /I_{N}^{2}$ is the zero-delay second-order correlation function dependent only on $M_{\omega_{0}/v}$ and $M_{2\omega_{0}/v}$ (see SI [43] for its exact form). Interestingly, it can uniquely exhibit Poissonian or super-Poissonian emission (*g* ^(2)^(0) ≥ 1) if the e-beam density is modified. This conclusion can be drawn from the positivity of the fluctuations and the fact that, if *g* ^(2)^(0) < 1, $\Delta I_{N}^{2}$ can assume an arbitrary negative value for strong enough coupling *β*_0_, as the correlation function is independent of its value.

**Figure 2:**
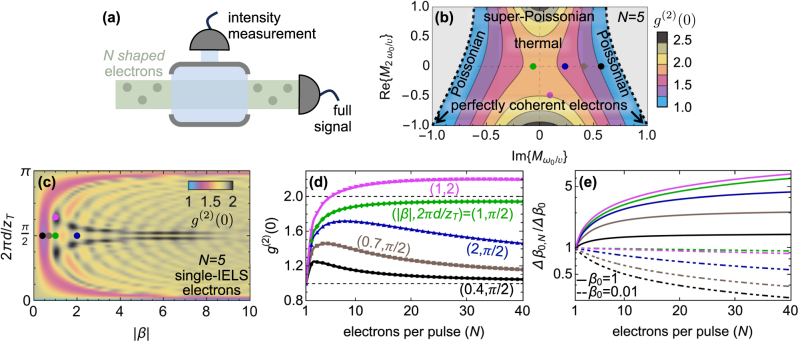
Intensity fluctuations for *N* modulated electrons. (a) *N*-electron modulated pulses emit light into a photonic mode. Its intensity is recorded together with its fluctuations by including all electron scattering events. (b) Second-order correlation function *g* ^(2)^(0) defining the statistics of the emitted light without post-filtering (see Eq. (4) and sketch in panel (a)) computed for *N* = 5 electrons. The electrons are assumed to undergo the same modulation yielding a coherence factor (CF) with imaginary $M_{\omega_{0}/v}$ and real $M_{2\omega_{0}/v}$ similarly to the CF after an IELS interaction (see Eq. (5)). The grey areas correspond to unphysical electron states and CF values leading to negative light intensity fluctuations. (c) Same as in (b) but for electrons emitting light after an IELS modulation of strength |*β*| and subsequent free propagation of *d* with respect to the Talbot distance $z_{\text{T}}=4\pi m_{e}v^{3}\gamma^{3}/\hbar \omega_{\text{L}}^{2}$ (see Eq. (5)). (d) Second-order correlation function as a function of the number of electrons *N* in each pulse. (e) Root mean square error in the estimate of *β*_0_ when measuring the light intensity emitted by pulses composed of *N* electrons, Δ*β*_0,*N*_, normalized to the error in the single-electron limit, Δ*β*_0_ (see Eq. (6)), for *β*_0_ = 0.01 (dashed lines) and *β*_0_ = 1 (solid lines). For illustrative purposes, continuous curves are obtained through interpolation of a discretized number of points, as shown in panel (d). The type of modulation in (d, e) is chromatically indicated by matching the colors of the curves to (|*β*|, 2*πd*/*z*_T_) coordinates in panel (c) and to values of the first and second CF in (b) (colored dots).

In Figure 2, we explore the statistics of the light generated by identically modulated electrons yielding equal CF without keeping track of the post-interaction electron energies, as shown in the sketch of Figure 2a. In particular, in Figure 2b we look at electron densities leading to a purely imaginary and real CF at *k* = *ω*_0_/*v* and 2*ω*_0_/*v*, respectively. We motivate this particular choice after inspecting the form of the CF given by an electron after a single IELS modulation at *ω*_L_ = *ω*_0_ and a macroscopic propagation *d* from the interaction zone

(5) $$\begin{array}{ll} M_{m\omega_{0}/v} & =i^{m}\mathrm{sign}{\left\{\sin\left(2\pi md/z_{\text{T}}\right)|\right\}}^{m}\\ & \;\times e^{-im\arg\left\{-\beta \right\}}J_{m}\left[4|\beta \sin\left(2\pi md/z_{\text{T}}\right)|\right] \end{array}$$

which can be calculated from Eq. (3) and the energy coefficients $c_{\ell }=J_{\ell }\left(2|\beta |\right)e^{i\ell \arg\left\{-\beta \right\}-2\pi i\ell^{2}d/z_{\text{T}}}$ using an envelope *ψ*_0_(*z*) spanning several optical cycles [25], [37], [58], [66]. The *J*_*ℓ*_(*x*) is the *ℓ*-th Bessel function, *d* is the distance of free propagation from the IELS interaction zone, $z_{\text{T}}=4\pi m_{e}v^{3}\gamma^{3}/\hbar \omega_{\text{L}}^{3}$ is the Talbot distance, and *β* is a complex coupling parameter analogous to *β*_0_ but incorporating phase and amplitude of the electric field produced by the laser scattering off a material boundary [12], [30], [57]. We observe that, already for *N* = 5 electrons (Figure 2b), a wide range of super-Poissonian light can be harnessed with specific electron modulations. For instance, an electron bunch with vanishing coherence is shown to lead to thermal light [63], [67] whereas electrons with unity CF yield Poissonian statistics. In Figure 2c, we show that these types of electron modulations can be directly reproduced through careful choice of the IELS parameters.

Interestingly, *g* ^(2)^(0) can also be tuned by varying the number of electrons under fixed IELS conditions, as shown in Figure 2d. This observation has important implications when estimating the coupling strength *β*_0_ from light intensity measurements. Specifically, when using a total number of electrons *K* = *RN*, divided into *R* pulses each containing *N* particles, the root mean square error associated with the estimation of *β*_0_, given by Δ*β*_0,*N*_ = |∂*β*_0_/∂*I*_*K*_|Δ*I*_*K*_, must be evaluated from the total measured intensity *I*_*K*_ and its fluctuations $\Delta I_{K}^{2}$ . Since each pulse corresponds to an independent measurement, both quantities are connected with *I*_*N*_ and $\Delta I_{N}^{2}$ by a multiplicative factor *R* (see the SI [43] for more details), leading to

(6) $$\Delta \beta_{0,N}=\Delta \beta_{0}\sqrt{\frac{1+I_{N}\left[g^{\left(2\right)}\left(0\right)-1\right]}{1+\left(N-1\right)|M_{\omega_{0}/v}{|}^{2}}},$$

where we have defined the shot-noise-limited single-electron root mean square error as $\Delta \beta_{0}=1/2\sqrt{K}$ . From Eq. (6), we observe that Poissonian emission combined with a high number of electrons per pulse improves the estimation by a factor of approximately $1/\sqrt{N}|M_{\omega_{0}/v}|$ . When *g* ^(2)^(0) deviates slightly from unity, this approximation remains valid for small values of *β*_0_, corresponding to low *I*_*N*_. Remarkably, a nearly tenfold reduction in the ratio Δ*β*_0,*N*_/Δ*β*_0_ can be achieved by a single IELS modulation stage in the estimation of a weak electron-mode coupling, as shown in Figure 2e.

A more complex situation is found for a general post-sample filtering function. In this case, the number representation *ρ*_*p*_ = ∑_*nn*′_ *ρ*_*p*,*nn*′_|*n*⟩⟨*n* ^′^| provides a clearer isolation of the role played by the input electron density matrix, which is otherwise obscured in the spatial dependence of the coherent states in Eq. (2). While again considering uncorrelated electrons and an initial vacuum state (*α* = 0), we calculate *ρ*_*p*,*nn*′_ from Eq. (2) through a combinatorial analysis leading to (see SI [43] for a detailed derivation)

(7) $$\rho_{p,nn^{\prime }}=\frac{1}{P_{F}}\;\;\;\underset{\begin{array}{c} k,k^{\prime },m\\ m^{\prime },\text{p},{\text{p}}^{\prime }\geq 0 \end{array}}{\sum }C_{m,m^{\prime },\text{p},{\text{p}}^{\prime }}^{\left(n,k,n^{\prime },k^{\prime }\right)}\int dq_{N}F\left(q_{N}\right)$$

$$\;\times \overset{N}{\underset{i=1}{\prod }}{PM}_{\omega_{0}\left(s_{i}^{\prime }-s_{i}\right)/v}^{i}\left[q_{i}+\frac{\omega_{0}}{2v}\left(s_{i}+s_{i}^{\prime }\right)\right],$$

where *s_i_ = m_i_ − m_i_′, s_i_′ = p_i_ − p_i_′*, while the *β*_0_-dependent coefficient $C_{m,m^{\prime },\text{p},{\text{p}}^{\prime }}^{\left(n,k,n^{\prime },k^{\prime }\right)}$ is defined in the SI [43] and its specific form is not of fundamental relevance to this work. The vectors **m**, **m**′, **p** and **p**′ are composed by positive integers and have dimension *N*. Interestingly, Eq. (7) condenses the electron dependence into the factor

(8) $${PM}_{k}^{i}\left(q\right)=\overset{\infty }{\underset{-\infty }{\int }}\;dz\;W_{e}^{i}\left(z,q\right)\;e^{ikz}$$

which we term projected coherence factor (PCF), as it plays a role similar to the CF when only a sub-set of scattering events are observed and it is defined through the electron Wigner function $W_{e}^{i}\left(z,q\right)=\int_{-\infty }^{\infty }\;dy\;\rho_{e}^{i}\left(z-y/2,z+y/2\right)\;e^{iqy}/2\pi$ [68] representing the quantum analogue of a classical phase-space density. Equation (8) reveals that when final energies are measured, the electron density involved in the interaction is only determined *a posteriori* through the post-filtering procedure. Specifically, the spatial frequencies that influence *ρ*_*p*,*nn*′_ are those arising from the Fourier transform along the propagation axis of the density obtained through the integration of the electron Wigner function over the finite momentum range set by *F*(**q**_*N*_). In Figure 1a of the SI [43], we illustrate the sub-cycle structuring of several such cuts of the Wigner function corresponding to an IELS-modulated electron, also measured through a reconstruction algorithm based on a double-IELS interaction scheme [22]. Reassuringly, when no post-filtering is applied, the momentum integral of the PCF coincides with the CF, namely, $M_{k}^{i}=\int_{-\infty }^{\infty }\;\text{d}q\;{PM}_{k}^{i}\left(q\right)$ , as is directly evident from the Wigner function definition.

### 2.2 Light-state purity and electron coherence

An ideal quantum state, unaffected by classical ensemble averages over initial conditions or mechanisms of decoherence, can be described by a pure state $|\psi_{p}〉=\sum_{n=0}^{\infty }\alpha_{p,n}|n〉$ and, equivalently, by the density matrix *ρ*_*p*_ = |*ψ*_*p*_⟩⟨*ψ*_*p*_|. Here, we aim to explore how electron coherence and post-filtering determine the final purity of the light.

First, we examine Eq. (2) in the case of uncorrelated electrons (although this assumption is not necessary for the following statement to hold) and observe that, if an infinitely precise post-filtering measurement with outcome ${\tilde{q}}_{N}$ , described by $F\left(q_{N}\right)∼\delta \left(q_{N}-{\tilde{q}}_{N}\right)$ , is performed, *ρ*_*p*_ becomes perfectly pure, provided the electron state is also pure, i.e., $\rho_{e}^{i}\left(z_{i},z_{i}^{\prime }\right)=\psi_{e}^{i}\left(z_{i}\right)\psi_{e}^{i*}\left(z_{i}^{\prime }\right)$ . In most experiments performed in SEM/TEM, the latter assumption is not met because electrons arrive at the sample at a time *t*_0,*i*_ that can incoherently fluctuate by Δ*t* ∼ 100 fs [53], [69], [70], [71]. However, since they have coherence times *σ*_*t*_ ∼ 5 fs spanning several optical cycles (*σ*_*t*_ *ω*_0_ ≫ 1), their PCF is not affected by the incoherent averaging at the spatial frequencies of interest for this work *k* = *mω*_0_/*v*, with *m* an integer number, therefore effectively providing the aforementioned purity condition (see SI [43] for a detailed proof). Thus, we conclude that, regardless of the specific form of the coherently modulated electron state, the determination of the final energies of all electrons guarantees a pure light state. However, such purity will be maintained over the spectral width ∼*ℏ*/Δ*t* ∼ 10 meV around *ω*_0_.

We now examine this result in the simple case of a single electron, for which Eq. (7) simplifies to the form (see SI [43])

(9) $$\begin{array}{ll} \rho_{p,nn^{\prime }} & =\frac{1}{P_{F}}〈n|\beta_{0}〉〈\beta_{0}|n^{\prime }〉\\ & \;\times \overset{\infty }{\underset{-\infty }{\int }}\;\text{d}q\;F\left(q\right)\;{PM}_{\omega_{0}\left(n^{\prime }-n\right)/v}\left[q+\omega_{0}\left(n+n^{\prime }\right)/2v\right]. \end{array}$$

**Figure 3:**
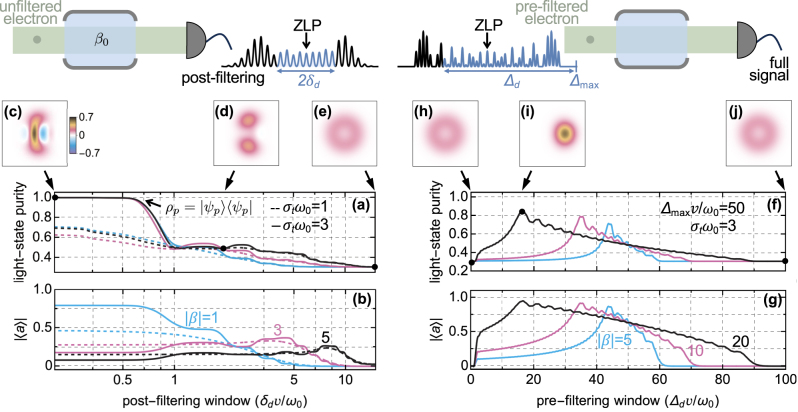
Properties of the light state generated by single electrons using energy post- or pre-filtering. An electron with an incoherent envelope of temporal width Δ*tω*_0_ ≫ 1 freely drifts over a negligible length with respect to the Talbot distance $z_{\text{T}}=4\pi m_{e}v^{3}\gamma^{3}/\hbar \omega_{\text{L}}^{2}$ , from a single IELS interaction of strength *β* and frequency *ω*_L_ = *ω*_0_ to couple with an optical mode with strength *β*_0_ = 1 initially in a vacuum state |0⟩⟨0|. After the interaction, the light state purity (a) and the absolute value of the average of the photonic destruction operator (b) are computed by considering the electrons with normalized coherence time *σ*_*t*_ *ω*_0_ = 1 (dashed lines) and 3 (solid lines) and longitudinal momentum in a window 2*δ*_*d*_ symmetric around the zero-loss peak (ZLP), as shown in the post-sample asymmetric spectrum above panels (c–e). (c–e) Photonic Wigner function after coupling with an electron with *σ*_*t*_ *ω*_0_ = 3 for the post-filtering windows δ_*d*_ *v*/*ω*_0_ = 0.01, 2, 15, respectively. (f, g) Same as (a, b) with *σ*_*t*_ *ω*_0_ = 3 but discarding the electrons outside the momentum range between Δ_max_ − Δ_*d*_ and Δ_max_ = 50*ω*_0_/*v* immediately after an IELS stage, as shown in the symmetric spectrum above panels (h–j), and without final energy post-filtering. (h–j) Photonic Wigner function corresponding to the pre-filtering windows at Δ_*d*_ *v*/*ω*_0_ = 0.01, 16.5, 100, respectively. In all panels, we use arg{−*β*} = 0.

In Figure 3a, we analyze the purity $\mathrm{Tr}\{\rho_{p}^{2}\}$ of the state in Eq. (9) for an electron with a coherent Gaussian envelope of standard deviation *σ*_*t*_ and incoherent ensemble distribution of width Δ*tω*_0_ ≫ 1 modulated through an IELS stage of laser frequency *ω*_L_ = *ω*_0_ and subsequently propagated over a distance *d* from the interaction zone, as done to obtain Eq. (5). As expected, the light-state purity approaches unity when the post-filtering window 2*δ*_*d*_, collected by the energy detector, is *δ*_*d*_ *v*/*ω*_0_ ≲ 0.5 as long as the electron coherence spans several optical cycles, while it stabilizes to the fully-mixed value $\sum_{n=0}^{\infty }\rho_{nn}^{2}$ , when the post-filtering window covers the entire electron spectrum. This result is in agreement with the form of the *m*-th order CF in Eq. (5), vanishing for *d*/*z*_T_ ∼ 0 and *m* ≠ 0, and the generated light state $\rho_{p,nn^{\prime }}=\left\langle n|\beta_{0}\right\rangle \left\langle \beta_{0}|n^{\prime }\right\rangle M_{\omega_{0}\left(n^{\prime }-n\right)/v}$ obtained from Eq. (9) in the *δ*_*d*_ → ∞ limit. Accordingly, the form of the photonic Wigner function [72], also showing negative values, represent a pure quantum state generated by an IELS electron for small *δ*_*d*_ and a phase-averaged coherent state where the entire spectrum is considered (see Figure 3c–e).

As we previously observed, in addition to enabling access to high-purity states, the combination of post-filtering and shaped electrons provides a means to probe time-varying signals with an electron density that depends on its final measured energy and that can be visualized through the energy cuts of the electron Wigner function (see Figure 1a in the SI [43]). An example of this is the average electric field $\left\langle \hat{E}\left(r\right)\right\rangle ={\vec{E}}_{0}\left(r\right)\left\langle \hat{a}\right\rangle +{\vec{E}}_{0}^{*}\left(r\right)\left\langle {\hat{a}}^{†}\right\rangle \propto |\left\langle \hat{a}\right\rangle |$ emitted by the electron into the light mode, which varies as a function of *δ*_*d*_ (see Figure 3b). This capability could be particularly significant for studying and controlling the dynamics in materials [73], [74] triggered by the same laser used to modulate the beam with sub-ps precision.

A similar phenomenon of enhanced time localization occurs when an energy filter, selecting a fixed momentum range starting from Δ_min_ = Δ_max_ − Δ_*d*_ and ending at Δ_max_ relative to the central momentum, is placed between the IELS modulation and the interaction with the sample (see Figure 1 and the rightmost sketch in Figure 3). Indeed, since the CF can be re-expressed in terms of the PCF of an electron without pre-filtering ${PM}_{k}^{\text{unf}}$ as

(10) $$M_{k}=\frac{1}{M_{0}}\overset{{\tilde{\Delta }}_{\max}}{\underset{{\tilde{\Delta }}_{\min}}{\int }}\;\;\;\text{d}q\;{PM}_{k}^{\text{unf}}\left(q+k/2\right)$$

with ${\tilde{\Delta }}_{\max}=\min\left\{\Delta_{\max},\Delta_{\max}-k\right\}$ and ${\tilde{\Delta }}_{\min}=\max\{\Delta_{\min},\Delta_{\min}-k\}$ , this procedure effectively corresponds to selecting an energy portion of *W*_*e*_(*z*, *q*), thereby influencing the involved electron density and its related quantities, such as the average electric field (see Figure 3g). The factor *M*_0_ represents the probability of pre-filtering and guarantees wave function normalization. The resulting enhanced electron coherence is also reflected by the light-state purity depicted in Figure 3f for an electron pre-filtered right after (*d* = 0) an IELS interaction. Here, we observe several maxima (with ∼0.86 the greatest value), each one for a given energy window *ℏ*Δ_*d*_ *v* and coupling strength *β* as well as a convergence to the mixed-state value for small and large Δ_*d*_. This behavior can be understood by examining the corresponding CF in the *σ*_*t*_ *ω*_0_ ≫ 1 limit, expressed as (see SI [43] for a detailed calculation):

(11) $$\begin{array}{ll} M_{m\omega_{0}/v} & =e^{-im\arg\left\{-\beta \right\}+2\pi im^{2}d/z_{\text{T}}}\\ & \;\times \frac{1}{M_{0}}\overset{\ell_{\max}}{\underset{\ell =\ell_{\min}}{\sum }}\;\;J_{\ell }\left(2|\beta |\right)J_{\ell +m}\left(2|\beta |\right)\;e^{4\pi im\ell d/z_{\text{T}}}, \end{array}$$

where *ℓ*_min_ = ⌊Δ_min_ *v*/*ω*_0_⌋ − min{0, *m*} + 1 and *ℓ*_max_ = ⌊Δ_max_ *v*/*ω*_0_⌋ − max{0, *m*}, and ⌊*x*⌋ denotes the floor function of *x*. This expression reveals a significant increase in electron coherence, surpassing the absolute maximum of $|M_{\omega_{0}/v}|∼0.58$ observed in bunched densities following an IELS interaction and a drift in free space [22], [30], [75]. For instance, with |*β*| ∼ 20, we achieve $|M_{\omega_{0}/v}|∼0.95$ for various values of *d*, including *d*/*z*_T_ ∼ 0 (see Figure 1b–d in the SI [43]). Given the macroscopic lengths on the centimeter scale required by standard energy filters to operate, such a case refers to an idealized scenario not experimentally achievable in a straightforward manner. However, at Talbot revivals and thus larger distances, depending on the coherence time and IELS strength, similar results could be achieved. In particular, optimal purity is achieved by filtering near the lobes of the IELS energy distribution, as in that region the electron density confines to a limited range in time (see Figure 1a in the SI [43]). Importantly, this type of strategy can also be used as an alternative approach to pulse compression [22], [23].

Despite this high coherence for low *m*, Eq. (11) vanishes for ⌊Δ_max_ *v*/*ω*_0_⌋ −⌊Δ_min_ *v*/*ω*_0_⌋≤ |*m*|, thereby limiting the light-state purity in a manner dependent on the electron-mode coupling *β*_0_. Finally, as previously demonstrated [30], *ρ*_*p*_ oscillates between a quasi-pure and a phase-averaged coherent state as the electron coherence is varied through Δ_*d*_ (see Figure 3h–j).

As expected, for nearly elastic attosecond imaging or diffraction experiments, it also becomes irrelevant if the filtering takes place before or after the sample. This is confirmed by the *k* → 0 limit of the integral in Eq. (10) that transforms to an integrated PCF over the collection range as it appears in Eqs. (7) and (9) for negligible *ω*_0_.

### 2.3 Natural synthesis of cat states by IELS electrons

**Figure 4:**
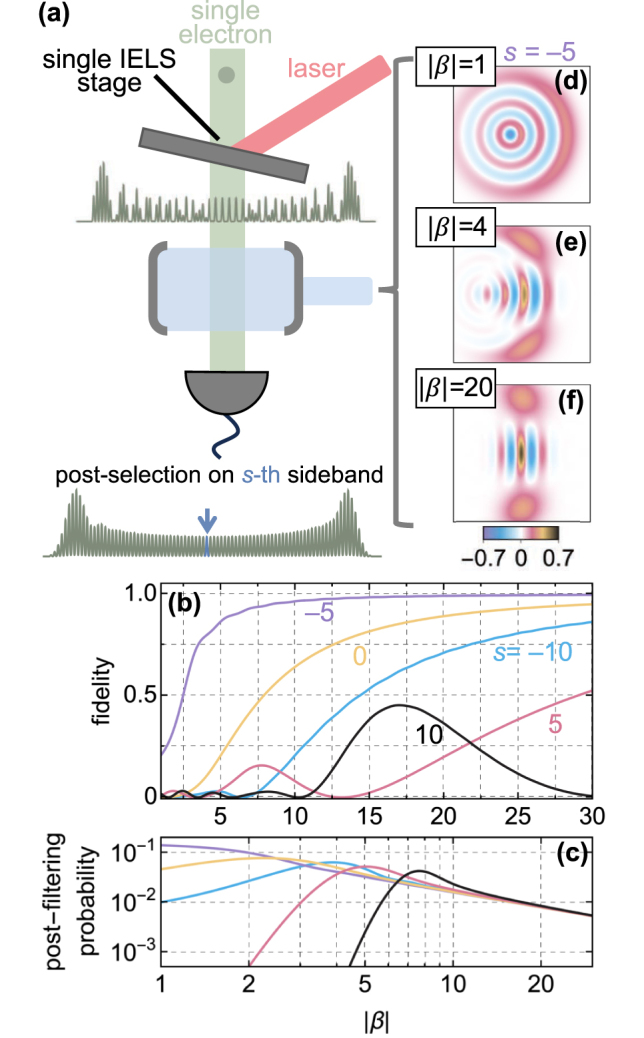
Natural formation of cat states after a single IELS stage. (a) Proposed scheme to produce high-purity cat states from an optical mode in a vacuum state involving a single IELS interaction of coupling parameter *β* and the post-filtering of the *s*-th sideband after spontaneous emission into the cavity with strength *β*_0_ = 2. (b) Overlap between the light state generated by an electron after passing through the stages sketched in (a) and a cat state with amplitude *χ* = −i*β*_0_e^iarg{−*β*}^ and relative phase *θ* = *sπ* + *π*/2 − 4|*β*| for different IELS couplings *β* and post-filtered sideband order *s*. (c) Post-filtering probabilities for the configurations reported in (b). (d–f) Post-interaction photonic Wigner function for *s* = −5 and |*β*| = 1, 4, 20. In all panels, we use arg{−*β*} = 0.

We now utilize the purity achieved through post-filtering performed around the *s*-th energy sideband in the high electron coherence limit of Figure 3a to examine the actual state of the generated light (see Figure 4a). Under these conditions, we can work in the *σ*_*t*_ *ω*_0_ ≫ 1 approximation for which the integral of the PCF in Eq. (9), taken around the post-filtering sideband, only selects specific energy coefficients from the modulated superposition and thus reduces to the simple product $c_{n+s}c_{n^{\prime }+s}^{*}$ (see SI [43] for a proof). This further confirms our previous result stating that any form of coherent electron energy shaping will yield *ρ*_*p*_ = |*ψ*_*p*_⟩⟨*ψ*_*p*_|. The expansion coefficients in number basis directly follow from it and read

(12) $$\alpha_{p,n}=\frac{〈n|\beta_{0}〉\;c_{n+s}}{\sqrt{\sum_{n=0}^{\infty }|〈n|\beta_{0}〉\;c_{n+s}{|}^{2}}}.$$

Equation (12) demonstrates that any target light state with finite support can be synthesized through appropriate shaping of the electron energy coefficients *c*_*ℓ*_. Intuitively, it predicts an average photon number that depends on *β*_0_ but can exceed the probability of spontaneous emission, $\beta_{0}^{2}$ . This effect arises from the post-filtering process, where only a subset of events is considered during the photon measurements, and is related to the weak value of a quantum observable [76].

In the special case of an electron immediately after a one-stage IELS interaction (*c*_*ℓ*_ = *J*_*ℓ*_(2|*β*|)e^i*ℓ*arg{−*β*}^), we find that, beyond a certain high value of |*β*|, the electron naturally forms an approximate version of a cat state, *α*_*p*,*n*_ ∝ ⟨*n*|*χ*⟩[1 + e^i*θ*^(−1)^*n*^], where *χ* = −i*β*_0_e^iarg{−*β*}^ and *θ* = *sπ* + *π*/2 − 4|*β*|. Taking this state as the target state $|\psi_{p}^{\text{targ}}〉=\sum_{n=0}^{\infty }\alpha_{p,n}^{\text{targ}}|n〉$ , we compute its overlap with |*ψ*_*p*_⟩ using the fidelity $|\left\langle \psi_{p}|\psi_{p}^{\text{targ}}\right\rangle {|}^{2}$ . Remarkably, this shows near-perfect generation under the condition ${\left(n_{\max}+s\right)}^{2}/2\ll |\beta |$ , determined by the first *n*_max_ coefficients required to accurately describe $|\psi_{p}^{\text{targ}}〉$ , which is itself set by the value of |*χ*| = *β*_0_ (see Figure 4b). The origin of this natural predisposition of IELS electrons to form cat states lies in the asymptotic behavior of the Bessel functions for large arguments. Specifically, the large argument approximation $J_{n+s}\left(2|\beta |\right)\approx e^{-i\theta /2}\left[{\left(-i\right)}^{n}+e^{i\theta }i^{n}\right]/\sqrt{4\pi |\beta |}$ reveals a superposition of two energy plane waves. Each of these components corresponds to the emission of a coherent state whose amplitude is shifted relative to the other by a *π* phase, exactly as required for the formation of a cat state. In more intuitive terms, in this regime, the sinusoidal modulation in phase space passes twice at fixed times through the region of small energy changes, leading to a superposition of electron density shifted by half a cycle. The high coupling strengths demanded in this approach have already been experimentally demonstrated with pulsed-laser interactions near a nanostructure [77] and in free space [78] as well as under continuous-wave seeding of a Si_3_N_4_ microresonator [13]. However, due to the large energy spread introduced by the |*β*| ≳ 10 IELS interaction, post-filtering probabilities are found to be ≲1 % (see Figure 4c) at fidelities ≳99 % (see Figure 4d–f).

### 2.4 On-demand quantum light generation by lateral IELS

**Figure 5:**
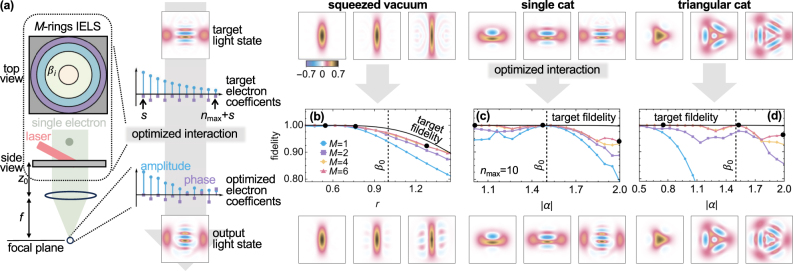
Lateral IELS patterning for electron energy coefficients optimization and quantum light generation. (a) Illustration of the steps employed for tailored synthesis of quantum light states. A set of electron energy amplitudes are obtained from Eq. (12) to approximate the first *n*_max_ = 10 coefficients of a given target photonic state $|\psi_{p}^{\text{targ}}〉$ and employed to optimize the design of the radial profile of the near-field used in an IELS stage composed of *M* concentric rings each with corresponding coupling constant *β*_*i*_. The most favorable design is supposed to provide the electron energy coefficients producing the optimal light state $|\psi_{p}^{\text{opt}}〉$ that maximizes the fidelity $|\left\langle \psi_{p}^{\text{targ}}|\psi_{p}^{\text{opt}}\right\rangle {|}^{2}$ . (b–d) Maximum achieved fidelity for *M* = 1, 2, 4, 6 concentric rings for different types of light states: a squeezed vacuum state with coefficient *r* (a), a cat state with real amplitude *α* and phase *θ* = *π*/2 (b), and a triangular cat state with real amplitude *α* and *θ* = 2*π*/3 (c). The photonic Wigner functions on the top row correspond to target states while the ones in the bottom to generated states in the configurations highlighted by the black circles in (b, c, d). A laser modulation frequency *ω*_L_ = 2*ω*_0_ and an electron-mode coupling strength *β*_0_ = 1 were used in (b) while *ω*_L_ = *ω*_0_ and *β*_0_ = 1.5 in (c, d).

The approach previously used to create a specific type of cat state can be generalized to a broader range of light states through Eq. (12) by accessing a wider set of electron energy coefficients *c*_*ℓ*_. Several schemes have been proposed to achieve such flexibility, primarily relying on either sequential combinations of IELS and free propagation stages [25] or focusing different lateral sections of an e-beam that has passed through a spatially dependent coupling coefficient *β*(**R**) [26]. A third strategy involves the use of shaping pulses composed of several harmonics [79], [80], however, its implementation would require a structure capable of sustaining strong IELS coupling strengths over a considerably broad spectral range, especially when operating in the visible regime. In this work, we adopt the scheme based on lateral field structuring whose capabilities are reported in Figure 5; however, a similar study could be conducted following the other methods.

As detailed in the SI [43], the energy coefficients forming the wave function near the focal point of a lens acting on an electron previously shaped by a near field divided into *M* equal-area circular sectors, each producing constant IELS coefficients *β*_*i*_ (see the modulation scheme in Figure 5a), are given by

(13) $$c_{\ell }=e^{-2\pi i\ell^{2}d/z_{\text{T}}}\overset{M}{\underset{i=1}{\sum }}J_{\ell }\left(2|\beta_{i}|\right)e^{i\ell \arg\left\{-\beta_{i}\right\}},$$

where now *d* = *z*_0_ + *f* is the sum of the lens’ separation from the IELS plane (*z*_0_) and the focal distance (*f*). We use an optimization algorithm based on a steepest descent routine (see SI [43] for details) to determine the set of coupling strengths *β*_*i*_, lens position *d*, and post-filtering sideband *s* that maximize the overlap of the generated state $|\psi_{p}^{\text{opt}}〉$ with a given target light state. This is achieved by repeatedly inserting Eqs. (13) into (12) (see Figure 5a). Specifically, the optimization process runs over $c_{s},\ldots ,c_{n_{\max}+s}$ while verifying that the inclusion of additional coefficients does not result in any significant changes.

As target states, we select the first *n*_max_ coefficients, which define a maximum achievable target fidelity (black solid lines in Figure 5b–d), for a squeezed vacuum with $\alpha_{p,2n}^{\text{targ}}\propto {\left(-\tanh r\right)}^{n}\sqrt{\left(2n\right)!}/2^{n}n!$ , a cat state $\alpha_{p,n}^{\text{targ}}\propto \left\langle n|\alpha \right\rangle \left[1+e^{i\theta }{\left(-1\right)}^{n}\right]$ , and a triangular cat state with $\alpha_{n}^{\text{targ}}\propto \left\langle n|\alpha \right\rangle \left[1+e^{in\theta }+e^{2in\theta }\right]$ (see the first row of photonic Wigner functions in Figure 5b–d). However, we remark that this method is applicable to any set of coefficients $\alpha_{p,n}^{\text{targ}}$ .

For the squeezed vacuum, we achieve fidelities of nearly 100 % for amounts of squeezing smaller than *β*_0_ by modulating the electron at twice the fundamental frequency (*ω*_L_ = 2*ω*_0_), which suppresses the emission of an odd number of photons for even *s*, simplifying the optimization. While this result is largely independent of the number of sectors for small *r*, when the average number of required photons exceeds $\beta_{0}^{2}$ , we observe a significant improvement in synthesizing the target state as *M* increases (see Figure 5b). For cat and triangular cat states, the ability of the coefficients in Eq. (13) to replicate $\alpha_{p,n}^{\text{targ}}$ improves dramatically with the addition of more circular sectors, raising the fidelity from below 80 % for *M* = 1 to nearly 100 % for *M* = 6 (see Figure 5c and d). Within the explored parameter range, the optimal IELS couplings are confined to the range 0 ≲ |*β*_*i*_| ≲ 15 (in Figure 2 in the SI [43], we report their values), while post-filtering probabilities range from 10 % to 0.1 %, depending on whether $\left\langle \psi_{p}^{\text{targ}}|\hat{n}|\psi_{p}^{\text{targ}}\right\rangle$ is smaller or larger than $\beta_{0}^{2}$ , respectively.

In Figure 5, we chose to run our optimization algorithm over the first *n*_max_ = 10 coefficients to ensure computational efficiency. This limitation is reflected in the target fidelity curve which does not reach 100 % in all cases and produces target states that are not exact, as is the case of target squeezed vacuum states with *r* > 1.

## 3 Discussion and concluding remarks

In this work, we have presented a compact theoretical framework that enables the study of the light state generated by the interaction of *N* pre-modulated electrons with a single optical mode, within a specific subset of scattering events selected by a final electron spectrometer (see Figure 1).

We have demonstrated that, without final energy filtering, the resulting light density matrix *ρ*_*p*_ can exhibit either Poissonian or super-Poissonian statistics due to inter-electron photon exchange. However, its purity is strongly constrained by the electron coherence, quantified by the absolute value of the coherence factor (CF) $M_{k}^{i}$ , i.e., the strength of the Fourier components of the single-electron density $\rho_{e}^{i}\left(z,z\right)$ (see Eq. (3)). Coherent *N*-electron pulses shaped by a single IELS stage are capable of producing light superradiantly while maintaining *g* ^(2)^(0) ∼ 1, an effect that can provide a means to probe small coupling strengths *β*_0_ from measurements of cathodoluminescence emission with a tenfold reduction in shot noise (see Figure 2e).

To enhance the CF to approximately 95 %, we proposed retaining only the electrons exiting a strong (|*β*| ∼ 20) IELS modulation with energies inside a specific window, which effectively compresses the e-beam temporally. The advantage of this scheme, compared to others that combine longitudinal [25] or later IELS interactions [26], is that it relies only on a single homogeneous IELS stage – a resource increasingly common in ultrafast TEM – and an energy filter, such as a Wien filter [81], placed before the sample rather than after, as in energy-filtered EELS measurements [82]. At optical frequencies, the optimal energy window is approximately 20 eV (see Figure 3f), making the filtering requirements less stringent than in such experiments. Using this practical scheme for a single electron, we have shown that coherent states with a purity of approximately 90 % can be generated (see Figure 3f).

We have also examined how *ρ*_*p*_, and the associated light properties, are influenced by electron modulation when post-filtering is applied to a specific kinetic energy window. Specifically, we found that electron coherence is now quantified by the projected coherence factor (PCF) (see Eq. (8)), where the electron density appearing in the CF is replaced by the electron Wigner function $W_{e}^{i}\left(z,q\right)$ integrated over a specific range of momenta. Since this range is selected *a posteriori*, this result demonstrates how different post-filtering windows can reveal information about a specimen probed through various sub-cycle density modulations. In terms of light state purity, we demonstrated that for any electron modulation yielding the energy coefficients *c*_*ℓ*_, a narrow post-filtering window produces a perfectly separable state, even under stochastic electron illumination with random times of arrival, provided the electrons have coherence times spanning several optical periods (see Figure 3a).

By leveraging this result, we have demonstrated several cases where quantum light can be harnessed using only a single IELS stage. We showed how cat states can be generated without lateral patterning of the IELS field or dispersive electron compression, achieving ∼100 % fidelity with probabilities exceeding 1 % (see Figure 4b and c). A practical realization of our proposed method should already be within reach of state-of-the-art experimental setups, combining near-unity quantum efficiency electron detectors and photonic chips that have proved strong IELS modulation with |*β*| ≈ 40 using microresonators operated at milliwatt optical powers [13]. In this context, photonic chips integrating multiple optical microresonators with light in- and out-coupling capabilities [83] offer the possibility to condense modulation and synthesis stages into a single photonic structure. While this approach requires sufficient suppression of inter-stage optical crosstalk, it inherently aligns the two interaction zones and eliminates differential mechanical noise between them.

Furthermore, to synthesize other types of light states, we proposed a scheme based on optimizing the *c*_*ℓ*_ coefficients produced by an IELS interaction composed of *M* concentric sectors (see Eq. (13)). Applying this approach to the generation of squeezed vacuum, cat, and triangular cat states, we demonstrated that *M* = 6 sectors are sufficient to achieve their production with ∼99 % fidelity and probabilities greater than 0.1 %, provided the required average number of photons remains close to the Poissonian spontaneous emission value $\beta_{0}^{2}$ . A first possible design aimed at the production of these states with reasonable fidelity might be realized using a two-sector (*M* = 2) plate with axial symmetry, as shown in the sketch of Figure 5a. Specifically, hybrid films composed of a dielectric layer coated with a metallic film of varying thickness could be uniformly illuminated to produce the desired amplitude and phase of *β*_1_ and *β*_2_ [26]. Electron-light coupling in similar geometries, consisting of apertures in gold films deposited on silicon membranes, has been shown to yield IELS strengths ∼1 for laser pulses with an average power of ∼10 mW illuminating areas of ∼0.1 µm^2^ [17]. Higher laser powers, constrained by the damage threshold of the materials, or smaller interaction areas are therefore required to generate |*β*_1_|, |*β*_2_| ≳ 3, enabling electron pulses optimized to emit quantum light containing a considerable number of photons (see Figure 2 in the SI [43]). In addition, careful consideration must be given to the separation *d* between modulation and interaction stages in relation to the Talbot distance (*z*_T_ ∼ 156 mm at $E_{0}^{e}=100$ keV energy and *ℏω*_0_ = 1.5 eV), which modulates the quadratic phase in Eq. (13). Since optimal distances are found to depend strongly on the specifics of the target photonic state, tunable quantum light sources intended to generate different states require scattering structures optimized over a broad range of frequencies and electron velocities, allowing for adjustment of *z*_T_ through the tuning of these two parameters.

In all analyzed cases, the creation of light states with strong quantum features, such as high squeezing or Wigner function negativity, requires a high average photon number, which in turn necessitates above-unity values of *β*_0_. Recent experiments with electrons passing extended structures of about ∼100 µm in length reported photon generation in a dielectric waveguide at an average coupling parameter of *β*_0_ ∼ 0.32 [40], and EELS at a hybrid metal-dielectric multilayer structure corresponding to *β*_0_ ∼0.99 [49]. In addition, several works have explored the fundamental limits constraining *β*_0_ in free-flying trajectories [84], [85], aiming to guide the search for higher coupling strengths, which are generally expected for longer interaction lengths [45]. Ultimately limited by electron diffraction, other proposals have suggested ponderomotive transverse confinement of electrons to mitigate beam expansion [86]. In the current optimization scheme (Figure 5a), the electron coefficients maintain the form reported in Eq. (13) only over a distance of approximately *λ*_*e*_/NA^2^, suggesting small numerical apertures at high energies such as NA ∼ 2 × 10^−4^ at $E_{0}^{e}=100$ keV. Alternatively, at lower kinetic energies and for larger numerical apertures, infrared plasmonic resonances with dimensions *D* on the order of tens of nanometers, such as those found in nanostructured two-dimensional materials [45], [87], [88], may be preferred. Since *D* ∼ 1/*k*_0_, this conclusion is further supported by the phase-matching condition *ω*_0_/*k*_0_ *v* ∼ 1, which suggests low electron velocities for small-sized structures. If the *c*_*ℓ*_ variation in the spontaneous-emission zone continues to represent a problem, its effect could be mitigated by explicitly incorporating the spatial dependence of the coefficients into the optimization process.

Another possibility to increase the bare coupling strength *β*_0_ is offered by the application of the optimization scheme to *N*-electron pulses, leveraging the superradiant enhancement to achieve an effective coupling strength of *Nβ*_0_. In practice, such implementation only requires Eq. (7) in the *σ*_*t*_ *ω*_0_ ≫ 1 and exact post-filtering limits, available in the SI [43], in order to compute the fidelity between target and emitted light states. The exploration of this approach is left for future work.

The analysis presented here marks a fundamental step toward a more complete understanding of *N*-electron emission into free space and photonic structures under general coupling conditions. Our findings pave the way for superradiance-enhanced cathodoluminescence measurements and the practical realization of tunable sources of complex quantum light in photonic devices, with potential applications in electron-based low-dose spectroscopy as well as in quantum metrology and imaging.

## Supplementary Material

Supplementary Material Details
