# Supplementary material for: Tunable quantum light by modulated free electrons
Source: Nanophotonics. 2025 May 22;14(11):1865–78. doi: 10.1515/nanoph-2025-0040 (PMC12133258; doi:10.1515/nanoph-2025-0040)
Supplement: Supplementary file 1 — Supplementary Material Details [file j_nanoph-2025-0040_suppl_001.pdf]

Tunable quantum light by modulated free electrons

– Supplementary Information –

Valerio Di Giulio,[1,2] Rudolf Haindl,[1,2] and Claus Ropers[1,2,*]

[1] *Department of Ultrafast Dynamics, Max Planck Institute for Multidisciplinary Sciences, D-37077 Göttingen, Germany*
[2] *4th Physical Institute - Solids and Nanostructures, Georg-August-Universität Göttingen, D-37077 Göttingen, Germany*

Contents

S1. Photonic density matrix after interaction with an N-electron pulse and successive post-sample energy filtering 1

S2. Number-state representation of the photonic density matrix: multi-electron Wigner function and the Projected Coherence Factor (PCF) 3
A. PCF for electrons with stochastic arrival times 4
B. Intensity fluctuations generated by N uncorrelated electrons 5
C. Estimation of the coupling strength β_0 through light intensity measurements 5

S3. Mode density matrix after the interaction with a single electron 5
A. Coherence factor of a modulated electron after energy filtering 6
B. Natural synthesis of cat states after a single unstructured inelastic electron-light scattering (IELS) interaction 7

S4. Modulation of energy coefficients through laterally-structured IELS interaction and their optimization 8
A. Energy coefficients in the interaction region 8
B. Optimization method 9

References 9

S1. PHOTONIC DENSITY MATRIX AFTER INTERACTION WITH AN N-ELECTRON PULSE AND SUCCESSIVE POST-SAMPLE ENERGY FILTERING

In this section, we want to evaluate the density matrix associated with a single optical mode of frequency ω_0 after the interaction with an electron beam composed by N relativistic electrons, all with central kinetic energies $E_0^e \gg \hbar\omega_0$ corresponding to a velocity $\mathbf{v} = v\hat{\mathbf{z}} = \hbar q_0\hat{\mathbf{z}}/\gamma m_e$, where $\gamma = 1/\sqrt{1-v^2/c^2}$, and the action of a post-sample energy filtering (post-filtering) performed by an electron spectrometer. In what follows, we will assume the interaction to happen along the propagation direction of the electron bunch crossing the transverse position $\mathbf{R}$ at some instant of time.

For the energies analyzed in this work, the electrons do not change considerably during the interaction time and as a consequence their dispersion relation $E_q = c\sqrt{m_e^2c^2 + \hbar^2q^2}$ can be expanded to retain only the first linear term as $E_q \approx m_e c^2 + E_0^e + \hbar\mathbf{v}\cdot(\mathbf{q}-\mathbf{q}_0)$. Under this assumption, also known as nonrecoil approximation, the scattering operator $\hat{\mathcal{S}}(t,-\infty)$ associated with the system dynamics can be worked out explicitly [1] and in second quantization it takes the form

$$\hat{\mathcal{S}}(\infty,-\infty) = \exp\left\{\mathrm{i}\hat{\chi} + \int_0^\infty d\omega\, g_\omega(\hat{b}_\omega^\dagger\hat{a}_\omega - \hat{b}_\omega\hat{a}_\omega^\dagger)\right\}. \quad \text{(S1)}$$

In Eq. (S1) $\hat{\chi}$ is an operator that accounts for nonresonant electron-electron coupling mediated by the electromagnetic environment and $\hat{b}_\omega = \sum_k \hat{c}_k^\dagger\hat{c}_{k+\omega/v}$ is the operator decreasing the electron wave vector of ω_0/v written in terms of the anticommuting fermionic operators $\hat{c}_k$ and $\hat{c}_k^\dagger$. The ladder operators $\hat{a}_\omega$ and $\hat{a}_\omega^\dagger$ respect the commuting relation $[\hat{a}_\omega, \hat{a}_{\omega'}^\dagger] = \delta(\omega-\omega')$. The coupling constant $g_\omega = \sqrt{\Gamma_{\rm EELS}(\omega)}$ dictating the rate of photons exchanged between electrons and the optical mode can be computed from the electron energy loss

---

*Electronic address: claus.ropers@mpinat.mpg.de

probability $\Gamma_{\rm EELS}(\omega)=(4e^2/\hbar)\int_{-\infty}^{\infty}dzdz'\cos[\omega(z-z')/v]{\rm Im}\{-G_{zz}(\mathbf{R},z,\mathbf{R},z',\omega)\}$ with the knowledge of the electromagnetic Green tensor $G(\mathbf{r},\mathbf{r}',\omega)$ [2]. We remind that the scattering operator in Eq. (S1) links the joint electron-mode state in the interaction picture in the infinite past $\rho(-\infty)$ to the one after the interaction is ended $\rho(\infty)$ through the relation $\rho(\infty)=\hat{\mathcal{S}}(\infty,-\infty)\rho(-\infty)\hat{\mathcal{S}}^\dagger(\infty,-\infty)$. In this work, we neglect the action of $\hat{\chi}$ on the electron bunch as it produces losses away from the optical mode frequencies and therefore could be filtered by energy spectrometer and because its effect influences only electrons which are temporally separated by few-fs whereas they are typically separated by hundreds of fs in bunches produced in transmission and scanning electron microscopes (SEM/TEM). Moreover, we consider the mode to have a high quality factor and to be well spectrally isolated from the other photonic resonances and having an electric field distribution $\vec{\mathcal{E}}_0(\mathbf{r})$.

Under these condtions, we can approximate the Green tensor as $G_{zz}(\mathbf{R},z,\mathbf{R},z',\omega)=\mathcal{E}_{0,z}(\mathbf{R},z)\mathcal{E}^*_{0,z}(\mathbf{R},z')/2\pi\hbar\omega_0(\omega^2-\omega_0^2+{\rm i}0^+)$, with 0^+ and infinitesimal positive number, which, plugged into the EELS probability allows us to rewrite the scattering operator as

$$\hat{\mathcal{S}}(\infty,-\infty)\approx\exp\Big\{\beta_0(\hat{b}\hat{a}^\dagger-\hat{b}^\dagger\hat{a})\Big\},$$

where, we have used the relation ${\rm Im}\{-1/(\omega^2-\omega_0^2+{\rm i}0^+)\}=\pi\delta(\omega-\omega_0)/2\omega_0$, we have defined the operators $\hat{b}=\hat{b}_{\omega_0}$, $\hat{a}=\lim_{\omega\to\omega_0}\hat{a}_\omega/\sqrt{f(\omega-\omega_0)}$, with $f(\omega-\omega_0)={\rm Im}\{-1/\pi(\omega-\omega_0+{\rm i}0^+)\}$, and we have introduced the single-mode coupling $\beta_0=(e/\hbar\omega_0)\big|\int_{-\infty}^{\infty}dz\,\mathcal{E}_{0,z}(\mathbf{R},z){\rm e}^{-{\rm i}\omega_0 z/v}\big|$. The commutation relation of the new bosonic operators can be computed through the limiting procedure $[\hat{a},\hat{a}^\dagger]=\lim_{\omega,\omega'\to\omega_0}[\hat{a}_\omega,\hat{a}^\dagger_{\omega'}]/\sqrt{f(\omega-\omega_0)f(\omega'-\omega_0)}=1$.

We now write the N-electron the density matrix before entering in the interaction zone as $\rho_e(-\infty)=\sum_{\mathbf{k}_N\mathbf{k}'_N}\rho_{e,\mathbf{k}_N,\mathbf{k}'_N}|\mathbf{k}_N\rangle\langle\mathbf{k}'_N|$ by expanding its components in terms of N-dimensional vector states $|\mathbf{k}_N\rangle=|k_1,\dots,k_N\rangle$ containing the longitudinal set of momenta of all electrons in the pulse. Then, we obtain the non-normalized post-interaction density matrix of the photonic mode, conditioned to the measurement $\mathbf{q}_N$ of the final momenta of all electrons, by projecting the evolved joint density matrix onto the state $|\mathbf{q}_N\rangle$, which reads

$$T^{\mathbf{q}_N}=\langle\mathbf{q}_N|{\rm e}^{\beta_0(\hat{b}\hat{a}^\dagger-\hat{b}^\dagger\hat{a})}\rho_e(-\infty)\otimes|\alpha\rangle\langle\alpha|\,{\rm e}^{\beta_0(\hat{b}^\dagger\hat{a}-\hat{b}\hat{a}^\dagger)}|\mathbf{q}_N\rangle, \tag{S2}$$

where we have also assumed the photonic mode to be previously coherently excited to the state $|\alpha\rangle={\rm e}^{-|\alpha|^2/2}\sum_n(\alpha^n/\sqrt{n!})|n\rangle$, i.e., we have taken $\rho(-\infty)=\rho_e(-\infty)\otimes|\alpha\rangle\langle\alpha|$. Eq. (S2) can be reduced in a very simple form by noticing that the real-space state $|\mathbf{z}_N\rangle=\sum_{\mathbf{k}_N}({\rm e}^{-{\rm i}\mathbf{k}_N\cdot\mathbf{z}_N}/L^{N/2})|\mathbf{k}_N\rangle$ (with L the quantization length) is an eigenstate of the electron destruction operator, i.e., $b|\mathbf{z}_N\rangle=j(\mathbf{z}_N)|\mathbf{z}_N\rangle=\left(\sum_{i=1}^N{\rm e}^{-{\rm i}\omega_0 z_i/v}\right)|\mathbf{z}_N\rangle$, by using the normalization condition $\langle\mathbf{z}_N|\mathbf{z}'_N\rangle=\delta(\mathbf{z}_N-\mathbf{z}'_N)$, and their completeness relation $\int d\mathbf{z}_N|\mathbf{z}_N\rangle\langle\mathbf{z}_N|=\sum_{\mathbf{k}_N}|\mathbf{k}_N\rangle\langle\mathbf{k}_N|=\mathcal{I}$. After some straightforward algebra involving the use of the property of the displacement operator ${\rm e}^{\theta a^\dagger-\theta^* a}|\alpha\rangle=|\alpha+\theta\rangle$ from Eq. (S2), we arrive at

$$T^{\mathbf{q}_N}=\frac{1}{L^N}\int d\mathbf{z}_N d\mathbf{z}'_N\,\rho_e(\mathbf{z}_N,\mathbf{z}'_N)\,{\rm e}^{{\rm i}\mathbf{q}_N\cdot(\mathbf{z}'_N-\mathbf{z}_N)}|\alpha+\beta_0 j(\mathbf{z}_N)\rangle\langle\alpha+\beta_0 j(\mathbf{z}'_N)|, \tag{S3}$$

where we have introduced the representation of the N-electron density matrix in space coordinates $\rho_e(\mathbf{z}_N,\mathbf{z}'_N)=\langle\mathbf{z}_N|\rho_e(-\infty)|\mathbf{z}'_N\rangle=\sum_{\mathbf{k}_N\mathbf{k}'_N}\rho_{e,\mathbf{k}_N\mathbf{k}'_N}{\rm e}^{{\rm i}(\mathbf{k}_N\cdot\mathbf{z}_N-\mathbf{k}'_N\cdot\mathbf{z}'_N)}/L^N$.

If we take our post-filtering procedure to be described by a function $F(\mathbf{q}_N)$ integrating over only a finite set of prescribed electron momenta, we can retrieve final photonic state through the prescription $\rho_p=\sum_{\mathbf{q}_N}F(\mathbf{q}_N)T^{\mathbf{q}_N}(\infty)/P_F=(L/2\pi)^N\int d\mathbf{q}_N F(\mathbf{q}_N)T^{\mathbf{q}_N}(\infty)/P_F$, now normalized by the probability of successful filtering probability $P_F\leq 1$, which is given by

$$\rho_p=\frac{1}{(2\pi)^N P_F}\int d\mathbf{q}_N F(\mathbf{q}_N)\int d\mathbf{z}_N d\mathbf{z}'_N\rho_e(\mathbf{z}_N,\mathbf{z}'_N)\,{\rm e}^{{\rm i}\mathbf{q}_N\cdot(\mathbf{z}'_N-\mathbf{z}_N)}|\alpha+\beta_0(\mathbf{z}_N)\rangle\langle\alpha+\beta_0(\mathbf{z}'_N)|. \tag{S4}$$

This form of the output light state can result quite useful when one is interested in the computation of photonic observables which can be written in terms of the normal ordered operators $a^{\dagger m}a^n$. For instance without post-filtering $[F(\mathbf{q}_N)=1]$, we can employ Eq. (S4) to compute $\langle a^{\dagger m}a^n\rangle={\rm Tr}\{a^n\rho_p a^{\dagger m}\}=\int d\mathbf{z}_N\rho_e(\mathbf{z}_N,\mathbf{z}_N)\beta_0^n(\mathbf{z}_N)\beta_0^{*m}(\mathbf{z}_N)$, which for $n=m=1$ reduces to the average number of emitted photons and agrees with the result in Ref. 1.

We can analyze two limits of Eq. (S4) depending on the shape of the filtering function: (*i*) no post-sample filtering $[F(\mathbf{q}_N)=1]$, where ρ_p only depends on the density $\rho_e(\mathbf{z}_N,\mathbf{z}_N)$ as already predicted in several other works [1, 3, 4]; (*ii*) for a separable N-electron state $\rho_e(\mathbf{z}_N,\mathbf{z}'_N)=\psi_e(\mathbf{z}_N)\psi^*_e(\mathbf{z}_N)$ and a filtering function well-peaked around a central value $\tilde{\mathbf{q}}_N$, the photonic density matrix becomes a separable state, i.e., it factorizes as $\rho_p=|\psi_p\rangle\langle\psi_p|$ with

$$|\psi_p\rangle=\frac{f}{(2\pi)^{N/2}P_F^{1/2}}\int d\mathbf{z}_N\psi_e(\mathbf{z}_N){\rm e}^{-{\rm i}\tilde{\mathbf{q}}_N\cdot\mathbf{z}_N}|\alpha+\beta_0(\mathbf{z}_N)\rangle, \tag{S5}$$

where $f = [\int d\mathbf{q}_N F(\mathbf{q}_N)]^{1/2}$. Equation (S5) states that a perfect energy post-filtering performed on a pure N-electron state yields a pure photonic state.

S2. NUMBER-STATE REPRESENTATION OF THE PHOTONIC DENSITY MATRIX: MULTI-ELECTRON WIGNER FUNCTION AND THE PROJECTED COHERENCE FACTOR (PCF)

We want now to isolate the contribution of the electron state to the formation of ρ_p. In order to do that, we study the number representation of the photonic density matrix $\rho_p = \sum_{n,n'=0}^{\infty} \rho_{p,nn'} |n\rangle\langle n'|$ for a generic N-electron state and post-filtering operation. With the aid of the multinomial equality $\left(\sum_{i=1}^N x_i\right)^n \left(\sum_{i=1}^N y_i\right)^k = \sum_{\mathbf{m},\mathbf{m}'\geq 0} C^{(n,k)}_{\mathbf{m}\mathbf{m}'} \prod_{i=1}^N x_i^{m_i} y_i^{m'_i}$, written in terms of the coefficient $C^{(n,k)}_{\mathbf{m}\mathbf{m}'} = (n; m_1, \dots, m_N)(k; m'_1, \dots, m'_N)$ and the multinomial factors $(n; m_1, \dots, m_N) = (n!/m_1! \dots m_N!)$, with the superscript (n,k) restricting (the coefficients are imposed to vanish otherwise) the sum over $\mathbf{m}$ ($\mathbf{m}'$) to the combinations satisfying $m_1 + \dots + m_N = n$ ($m'_1 + \dots + m'_N = k$), we rewrite the components of Eq. (S4) for $\alpha = 0$ as

$$\rho_{p,nn'} = \frac{1}{P_F} \sum_{\substack{k,k',\mathbf{m} \\ \mathbf{m}',\mathbf{p},\mathbf{p}'\geq 0}} \mathcal{C}^{(n,k,n',k')}_{\mathbf{m}\mathbf{m}'\mathbf{p}\mathbf{p}'} \int d\mathbf{q}_N F(\mathbf{q}_N) PM_{\omega_0(\mathbf{m}'-\mathbf{m}+\mathbf{p}-\mathbf{p}')/v} \left[\mathbf{q}_N + \frac{\omega_0}{2v}(\mathbf{m} - \mathbf{m}' + \mathbf{p} - \mathbf{p}')\right], \tag{S6}$$

where we have introduced the β_0-dependent combinatorial coefficient

$$\mathcal{C}^{(n,k,n',k')}_{\mathbf{m}\mathbf{m}'\mathbf{p}\mathbf{p}'} = [(-2)^{-(k+k')} \beta_0^{2(k+k')+n+n'} / \sqrt{n!n'!}k!k'!] C^{(n+k,k)}_{\mathbf{m}\mathbf{m}'} C^{(n'+k',k')}_{\mathbf{p}\mathbf{p}'}.$$

Equation (S6) shows that the N-electron density matrix appears only in terms of the projected coherence factor (PCF)

$$PM_{\mathbf{k}_N}(\mathbf{q}_N) = \int d\mathbf{z}_N W_e(\mathbf{z}_N, \mathbf{q}_N)\, \mathrm{e}^{\mathrm{i}\mathbf{k}_N\cdot\mathbf{z}_N} \tag{S7}$$

defined through the quantum generalization of the classical phase-space density for the multi-electron state: the N-electron Wigner function

$$W_e(\mathbf{z}_N, \mathbf{q}_N) = \frac{1}{(2\pi)^N} \int d\mathbf{y}_N \rho_e(\mathbf{z}_N - \mathbf{y}_N/2, \mathbf{z}_N + \mathbf{y}_N/2)\, \mathrm{e}^{\mathrm{i}\mathbf{q}_N\cdot\mathbf{y}_N}. \tag{S8}$$

The term PCF is inspired by the coherence factor (CF) defined in several other works [1, 5, 6] for a single particle in an electron bunch of uncorrelated electrons as the Fourier transform of the density $M_k = \int_{-\infty}^{\infty} dz\, \rho_e(z,z)\, \mathrm{e}^{\mathrm{i}kz}$, to which it reduces when no post-filtering is performed. This last statement can be simply verified by integrating Eq. (S7) over $\mathbf{q}_N$ and by using the property of the Wigner function $\int d\mathbf{q}_N W_e(\mathbf{z}_N, \mathbf{q}_N) = \rho_e(\mathbf{z}_N, \mathbf{z}_N)$. The PCF contains the Fourier components of the Wigner function for a given post-selected longitudinal momentum window. In Fig. (S1a), we report some cuts of the Wigner function for a single electron integrated over an infinitesimal momentum window [see Eq. (S10a) below].

In the case of uncorrelated electrons, the density matrix can be written as the product of one-electron density matrices $\rho_e(\mathbf{z}_N, \mathbf{z}'_N) = \prod_{i=1}^N \rho_e^i(z_i, z'_i)$, which in turn, given Eq. (S7) and Eq. (S8), leads to the factorization of the PCF $PM_{\mathbf{k}_N}(\mathbf{q}_N) = \prod_{i=1}^N PM^i_{k_i}(q_i)$. Moreover, in the special case of pure electron states $\rho_e^i(z,z') = \psi_e^i(z)\psi_e^{i*}(z')$ and of a post-filtering window narrow around the vector $\tilde{\mathbf{q}}_N = \omega_0\mathbf{s}/v$, with $\mathbf{s}$ and N-dimensional vector of integer numbers, the state in Eq. (S6) purifies and the state coefficients of Eq. (S5) become

$$\alpha_{p,n} = \frac{f}{P_F^{1/2}} \sum_{k,\mathbf{m},\mathbf{m}'\geq 0} [C^{(n+k,k)}_{\mathbf{m}\mathbf{m}'}(-2)^{-k}\beta_0^{2k+n}/\sqrt{n!}k!] \prod_{i=1}^N \int_{-\infty}^{\infty} \frac{dz}{\sqrt{2\pi}} \psi_e^i(z) \mathrm{e}^{\mathrm{i}\omega_0(m'_i - m_i + s_i)z/v}.$$

The previous expression assumes a simple but useful form for the application of the modulation optimization algorithm presented in Sec. S4 and in the main text applied to multiple electrons having a wave packet with infinite coherence time $\sigma_t = L/v$ (where $L \to \infty$ at the end of the calculations) of the type $\psi_e^i(z) = \sum_{\ell=-\infty}^{\infty} c_\ell^i\, \mathrm{e}^{\mathrm{i}\ell\omega_0 z/v}/\sqrt{L}$. Indeed, by taking $F(\mathbf{q}) = (2\pi/L)^N \delta(\mathbf{q} - \tilde{\mathbf{q}}_N)$ and such type of the state, the coefficients $\alpha_{p,n}$ in the number representation $|\psi_p\rangle = \sum_{n=0}^{\infty} \alpha_{p,n}|n\rangle$ becomes

$$\alpha_{p,n} = \frac{1}{P_F^{1/2}} \sum_{k,\mathbf{m},\mathbf{m}'\geq 0} [(-2)^{-k}\beta_0^{2k+n} C^{(n+k,k)}_{\mathbf{m}\mathbf{m}'}/\sqrt{n!}k!] \prod_{i=1}^N c^i_{m_i+s_i-m'_i}. \tag{S9}$$

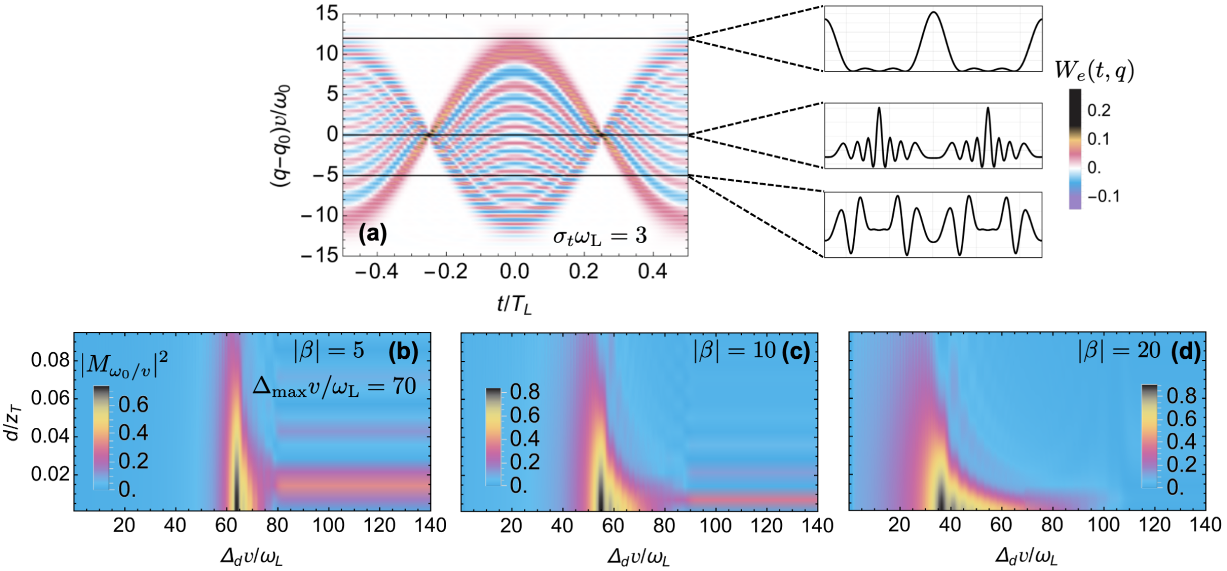

FIG. S1: **Coherence of modulated electrons.** **(a)** Momentum-time correlation expressed by the electron Wigner function [see Eq. (S10a) with $t = z/v$ and $T_L = 2\pi/\omega_L$] with coherence time $\sigma_t\omega_L = 3$ right after a IELS modulation $c_\ell = J_\ell(2|\beta|)e^{i\ell \arg\{-\beta\}}$ of interaction strength $|\beta| = 5.7$ at laser frequency $\omega_L = \omega_0$. The cuts along the time axis show a well-defined sub-cycle modulation for fixed normalized momentum. **(b-d)** Absolute squared value of the CF $|M_{\omega_0/v}|^2$ for an electron after an IELS interaction, free propagation of a distance d [appending a phase $-2\pi\ell^2 d/z_T$ to the c_ℓ used in (a)], and an energy filtering stage [see Eq. (S17)] selecting only a portion of longitudinal momenta Δ_d for $|\beta| = 5$ (b), 10 (c), and 20 (d). The maximum values found are $|M_{\omega_0/v}|^2 \sim 0.74$, 0.84, and 0.91 respectively. In all panels $\arg\{-\beta\} = 0$.

A. PCF for electrons with stochastic arrival times

In SEM/TEM, the coherence time of each electron σ_t is typically several times smaller than its classical (or incoherent) uncertainty Δ_t acquired by the electron ensemble through the random fluctuations of the electron source and of the instrumentation. Such fluctuations are responsible for the random arrival times at which the electrons reach the sample plane. In order to explore the consequences linked to this incoherent portion of the N-electron state, we study uncorrelated electrons with density matrix $\rho_e^i(z,z') = \int_{-\infty}^{\infty} dz_0 P(z_0)\psi_e^i(z,z_0)\psi_e^{i*}(z',z_0)$ defined by a classical distribution $P(z_0)$ of longitudinal planes z_0 that the electron crosses at $t = 0$ and a coherent wave function $\psi_e^i(z,z_0)$. For instance, in the case of an electron modulated by a IELS interaction at frequency ω_L, for which the wave packet takes the general form $\psi_e^i(z,z_0) = \psi_0^i(z,z_0)\sum_{\ell=-\infty}^{\infty} c_\ell^i e^{i\ell\omega_L z/v}$ assuming a Gaussian envelope $\psi_0^i(z,z_0) = e^{-(z-z_0)^2/4v^2\sigma_t^2 + iq_0 z}/(2\pi v^2\sigma_t^2)^{1/4}$, with the coefficients c_ℓ^i which depends on the form of modulation [see for instance Eq. (S19)] and they are chosen to ensure the normalization condition $\int_{-\infty}^{\infty} dz|\psi_e^i(z,z_0)|^2 = 1$.

By plugging this density matrix in the definition of the Wigner function and the PCF, one obtains

$$W_e^i(z,q) = \frac{1}{\pi}\sum_{\ell,\ell'} c_\ell c_{\ell'}^* \, e^{-[q-q_0-(\ell+\ell')\omega_L/2v]^2 2v^2\sigma_t^2} e^{i(\ell-\ell')\omega_L z/v}\int_{-\infty}^{\infty} dz_0 P(z_0) e^{-(z-z_0)^2/2v^2\sigma_t^2}, \tag{S10a}$$

$$PM_k^i(q) = \sqrt{\frac{2v^2\sigma_t^2}{\pi}}\sum_{\ell,\ell'=-\infty}^{\infty} c_\ell c_{\ell'}^* \, e^{-[q-q_0-(\ell+\ell')\omega_L/2v]^2 2v^2\sigma_t^2} e^{-[(\ell-\ell')\omega_L/v+k]^2 v^2\sigma_t^2/2} \tag{S10b}$$

$$\times\int_{-\infty}^{\infty} dz_0 P(z_0) e^{i[(\ell-\ell')\omega_L/v+k]z_0}.$$

For momenta $k = m\omega_0/v$ and modulation frequency $\omega_L = \omega_0$, with m and integer, as required by the computation of Eq. (S6), and in the limit of $\sigma_t\omega_0 \gg 1$, the effect of the exponential in the first line of Eq. (S10b) and the one arising from the incoherent integral $\int_{-\infty}^{\infty} dz_0 P(z_0) e^{i[(\ell-\ell')\omega_0/v+k]z_0}$ is equivalent, i.e., to enforce the $m = \ell' - \ell$ condition. Indeed, for a Gaussian ensemble of arrival times $P(z_0) = e^{-z_0^2/2v^2\Delta t^2}/\sqrt{2\pi v^2\Delta t^2}$ where typically $\Delta t\omega_0 \gg \sigma_t\omega_0 \gg 1$, such integral gives $e^{-[(\ell-\ell')\omega_0/v+k]^2 v^2\Delta t^2/2}$. Therefore in this regime, ρ_p can be equivalently evaluated by directly starting from the pure single-electron density matrix $\rho_e^i(z,z') = \psi_e^i(z)\psi_e^{i*}(z')$ disregarding the incoherent average on z_0. However, the purity of light states generated by electrons with coherence times smaller than the mode optical cycle will be strongly affected by it.

B. Intensity fluctuations generated by N uncorrelated electrons

From Eq. (S4), we can compute the amount of light emitted $I_N = \langle \hat{a}^\dagger \hat{a}\rangle \equiv \langle \hat{n}\rangle$ and its fluctuations $\Delta I_N = \langle \hat{n}^2\rangle - \langle \hat{n}\rangle^2$ by N modulated electrons with random times of arrival and large coherence times. This is easily done by utilizing the properties of the coherence state to obtain

$$I_N = \beta_0^2 \left[N + \sum_{i\neq i'=1}^{N} M^i_{\omega_0/v} M^{i'*}_{\omega_0/v} \right], \tag{S11}$$

$$\Delta I_N^2 / I_N = 1 + I_N[g^{(2)}(0) - 1], \tag{S12}$$

where we have introduced the zero-delay second-order autocorrelation function $g^{(2)}(0) = \langle \hat{a}^{\dagger 2}\hat{a}^2\rangle / I_N^2 = (\beta_0^4/I_N^2)\sum_{\mathbf{m},\mathbf{m}'\geq 0} C^{(2,2)}_{\mathbf{m},\mathbf{m}'} \prod_{i=1}^N M^i_{\omega_0(m_i'-m_i)/v}$. Super-Poissonian statistics is observed for electrons modulated such that $g^{(2)}(0) > 1$. Reassuringly, by exploiting the property $\sum_{\mathbf{m}\geq 0} \delta_{m_1+\cdots+m_N,n}(n; m_1, \dots, m_N) = N^n$, we recover a Poissonian emission $I_N = \Delta I_N^2$ in the limit of classical electrons for which $M_{\omega_0 m/v} = 1$ for any m. Interestingly, since ΔI_N^2 and I_N must be real positive numbers and $g^{(2)}(0)$ does not depend on β_0, we conclude that CF yielding $g^{(2)}(0) < 1$ would lead to arbitrary negative fluctuations for an increasing spontaneous emission coupling thus corresponding to unphysical electron states. We remark that, for identically modulated electrons, we have $I_N = \beta_0^2 N[1 + (N-1)|M_{\omega_0/v}|^2]$.

C. Estimation of the coupling strength β_0 through light intensity measurements

We are interested in evaluating the root mean square error associated with the estimate of the coupling strength β_0, based on measurements of the light intensity generated by R independent N-electron pulses, corresponding to a total number of electrons $K = RN$. To this end, we define K ladder operators $\hat{a}_i$, each acting on a separate system, to emulate the effect of statistically independent measurements. By following similar steps to those used in the previous section, we can calculate the total light intensity $I_K = \sum_{i=1}^R \langle \hat{a}_i^\dagger \hat{a}_i\rangle = R I_N$ and its fluctuations $\Delta I_K^2 = \sum_{i,j=1}^R \langle \hat{a}_i^\dagger \hat{a}_i \hat{a}_j^\dagger \hat{a}_j\rangle - I_K^2 = R\Delta I_N^2$, with the averages evaluated from the light state in Eq. (S4), without post-sample energy filtering.

Then, the root mean square error $\Delta\beta_{0,N} = |\partial\beta_0/\partial I_K|\Delta I_K$ follows from Eqs. (S11, S12), and reads

$$\Delta\beta_{0,N} = \Delta\beta_0 \sqrt{\frac{1 + I_N[g^{(2)}(0) - 1]}{1 + (N-1)|M_{\omega_0/v}|^2}}, \tag{S13}$$

where, to arrive at Eq. (S13), we have defined the shot-noise-limited single-electron root mean square error $\Delta\beta_0 = 1/2\sqrt{K}$ and assumed that all electrons are modulated in the same way, i.e., have the same CF. In the limit of many electrons per pulse ($N \gg 1$) and nonzero coherence, we obtain $\Delta\beta_{0,N}/\Delta\beta_0 \approx \sqrt{1 + N^2|M_{\omega_0/v}|^2\beta_0^2[g^{(2)}(0) - 1]}/\sqrt{N}|M_{\omega_0/v}|$, which predicts an improvement in the β_0 estimation by a factor $1/\sqrt{N}|M_{\omega_0/v}|$ in the case of Poissonian emission ($g^{(2)}(0) \approx 1$) and small β_0.

S3. MODE DENSITY MATRIX AFTER THE INTERACTION WITH A SINGLE ELECTRON

When only a single modulated electron is involved, we have $C^{(n+k,k)}_{mm'} = C^{(n'+k',k')}_{pp'} = 1$ which directly allows us to rewrite Eq. (S6) as

$$\rho_{p,nn'} = \frac{1}{P_F} \langle n|\beta_0\rangle\langle\beta_0|n'\rangle \int_{-\infty}^{\infty} dq\, F(q)\, PM_{\omega_0(n'-n)/v}[q + \omega_0(n+n')/2v]. \tag{S14}$$

For a post-filtering close to the m-th sideband, we can take $F(q)$ to vanish everywhere apart from the segment $q_0 + s\omega_0/v + [-\delta_d, \delta_d]$, that, plugged into Eq. (S14) with the electron state used to obtain Eq. (S10b) modulated at frequency $\omega_\mathrm{L} = \omega_0$, and with a Gaussian incoherent ensemble, yields

$$\rho_{p,nn'} = \frac{1}{2P_F} \langle n|\beta_0\rangle\langle\beta_0|n'\rangle \sum_{\ell,\ell'=-\infty}^{\infty} c_\ell c_{\ell'}^*\, \mathrm{e}^{-[\ell-\ell'+n'-n]^2\omega_0^2(\sigma_t^2+\Delta t^2)/2} \tag{S15}$$
$$\times \left\{ \mathrm{Erf}\left[\sqrt{2}\omega_0\sigma_t(\delta_d v/\omega_0 + x_0)\right] + \mathrm{Erf}\left[\sqrt{2}\omega_0\sigma_t(\delta_d v/\omega_0 - x_0)\right] \right\},$$

where we have made used of the integral $\int_{-\delta_d}^{\delta_d} dx \exp\{-(x-x_0)^2\sigma^2\} = \sqrt{\pi/4\sigma^2}\{\mathrm{Erf}[(\delta_d - x_0)\sigma] + \mathrm{Erf}[(\delta_d + x_0)\sigma]\}$ with $x_0 = (\ell+\ell')/2 - [(n+n')/2+s]$ and $\sigma = \sqrt{2}\omega_0\sigma_t$. The function $\mathrm{Erf}(x) = (2/\sqrt{\pi})\int_0^x dz\, \mathrm{e}^{-z^2}$ is the error function. In the limit $\delta_d\sigma_t v \gg |x_0|$, one can verify that the state only depends on the CF $M_{\omega_0(n'-n)/v} = \sum_{\ell,\ell'=-\infty}^{\infty} c_\ell c_{\ell'}^* \exp\{-(\ell-\ell'+n'-n)^2\omega_0^2(\sigma_t^2+\Delta t^2)/2\}$ and $M_{\omega_0(n'-n)/v} \approx \sum_{\ell=-\infty}^{\infty} c_\ell c_{\ell+n'-n}^*$ for $\sqrt{\sigma_t^2+\Delta t^2}\omega_0 \gg 1$. In the opposite limit of precise sideband determination ($\delta_d\sigma_t v \ll 1$), by using the expansion $\mathrm{Erf}[\sigma(x+x_0)] + \mathrm{Erf}[\sigma(x-x_0)] \approx (4\sigma x/\sqrt{\pi})\exp\{-x_0^2\sigma^2\}$, we obtain the separable state $\rho_p = |\psi_p\rangle\langle\psi_p|$ if $\Delta t\omega_0 \ll 1$ or $\sigma_t\omega_0 \gg 1$.

From Eq. (S15), in the case of large coherence time ($\sigma_t\omega_0 \gg 1$), we can approximate $\mathrm{Erf}(\sigma[x+x_0]) \approx \theta(x+x_0) - \theta(x-x_0)$ [where $\theta(x)$ is the Heaviside step function], which for a post-filtering procedure not overlapping with other sidebands ($\delta_d v/\omega_0 < 1$) leads to the pure state [in agreement with Eq. (S9)] with expansion coefficients

$$\alpha_{p,n} = \frac{1}{P_F^{1/2}}\langle n|\beta_0\rangle\, c_{n+s}, \tag{S16}$$

where $P_F = \sum_{n=0}^{\infty} |\langle n|\beta_0\rangle c_{n+s}|^2$. It is interesting to notice that, since the normalization constant $P_F \le 1$ and the average number of photons respects the inequality $\sum_{n=0}^{\infty} n|\alpha_{p,n}|^2 \le \beta_0^2/P_F$, its value can assume values larger than the number of photons one would measure without post-filtering the electron energy. Meaningfully, because P_F represents the probability of such post-filtering procedure, the larger the deviation from the average, the bigger the time needed to acquire sufficient statistics. An evident constraint arising from Eq. (S16), it is related to the asymptotic behavior of $\alpha_{p,n}$. Indeed, since the electron coefficients are normalized ($\sum_{\ell=-\infty}^{\infty}|c_\ell|^2 = 1$), the limit $\lim_{n\to\infty}\alpha_{p,n}/\langle n|\beta_0\rangle = 0$ needs to be satisfied for the electron state to be physical. This restricts the possible syntheses to states which have any type of coefficient over a finite set of $\alpha_{p,n}$, for instance by choosing all values from $\alpha_{p,0}$ to $\alpha_{p,n_{\rm max}}$, and then which decay faster than the components of a coherent state. Due to its generality, this procedure allows for almost perfect generation of any type of state as long as its average number of photons is $\ll n_{\rm max}$.

A. Coherence factor of a modulated electron after energy filtering

We want now to analyze the CF $M_k = \int_{-\infty}^{\infty} dz \rho_e(z,z)\, \mathrm{e}^{\mathrm{i}kz}/M_0$ (the factor M_0 has been added to account for an non-normalized electron density matrix), for a modulated Gaussian electron at the exit of an energy filter [7]. In order to do it, we firstly need to compute the electron state after the filtering process which we write by taking the Fourier components of $\rho_e(z,z') = \int_{-\infty}^{\infty} dq dq'\, \rho_e(q,q')\, \mathrm{e}^{\mathrm{i}qz-\mathrm{i}q'z'}/4\pi^2$ and then by multiplying them by a function $\mathcal{W}(q)$ representing the energy-filtering process.

It is convenient to evaluate the CF through the expression $M_k = \int_{-\infty}^{\infty} dq\, \rho_e(q,q+k)\mathcal{W}(q)\mathcal{W}(k+q)/2\pi M_0$, which, for $\mathcal{W}(q) = \theta(q-q_0-\Delta_{\rm max}+\Delta_d)\theta(\Delta_{\rm max}-q+q_0)$, with $\Delta_d > 0$, selecting longitudinal momenta in the range $[\Delta_{\rm max}-\Delta_d, \Delta_{\rm max}]$ around q_0 for the electron state used to write Eq. (S15), gives

$$\begin{aligned} M_k =& \frac{1}{2M_0}\sum_{\ell,\ell'} c_\ell c_{\ell'}^* \mathrm{e}^{-(\ell-\ell'+vk/\omega_0)^2(\sigma_t^2+\Delta t^2)\omega_0^2/2}, \quad \text{for } \Delta_d > |k| \\ &\times\Big\{\mathrm{Erf}\big[(2\Delta_{\rm max} - 2k_+ - k_{\ell+\ell'})\sigma_t\omega_0/\sqrt{2}\big] + \mathrm{Erf}\big[(k_{\ell+\ell'} + 2k_- - 2\Delta_{\rm max} + 2\Delta_d)\sigma_t\omega_0/\sqrt{2}\big]\Big\}, \end{aligned} \tag{S17}$$

where $k_{\ell+\ell'} = (\ell+\ell')\omega_0/v - k$, $k_+ = \max\{0,k\}$, and $k_- = \min\{0,k\}$, and 0 otherwise. In the $\sigma_t\omega_0 \gg 1$ limit, by again approximating the error functions as done in the previous section, the CF of Eq. (S17) at $k = m\omega_0/v$, for $\ell_{\rm max} = \lfloor\Delta_{\rm max}v/\omega_0\rfloor - \max\{0,m\}$ and $\ell_{\rm min} = \lfloor(\Delta_{\rm max}-\Delta_d)v/\omega_0\rfloor - \min\{0,m\} + 1$, where $\lfloor x\rfloor$ is the floor function of x, reduces to

$$M_{\omega_0 m/v} = \frac{1}{M_0}\sum_{\ell=\ell_{\rm min}}^{\ell_{\rm max}} c_\ell c_{\ell+m}^*. \tag{S18}$$

Interestingly, this filtering procedure can lead to CF of larger absolute values than the unfiltered version but the number of included energy coefficients needs to be larger than m for the CF to do not vanish, i.e., $\lfloor\Delta_{\rm max}v/\omega_0\rfloor - \lfloor(\Delta_{\rm max}-\Delta_d)v/\omega_0\rfloor \ge |m|+1$ [see Fig. (S1b-c)]. Pre-sample filtering is intimately connected to post-filtering as the CF of a filtered electron can be rewritten in terms of the PCF of an unfiltered electron $PM_k^{\rm unf}(q+k/2) = \rho_e(q,q+k)/2\pi$ as $M_k = \int_{-\infty}^{\infty} dq\mathcal{W}(q)\mathcal{W}(q+k)PM_k^{\rm unf}(q+k/2)/M_0$.

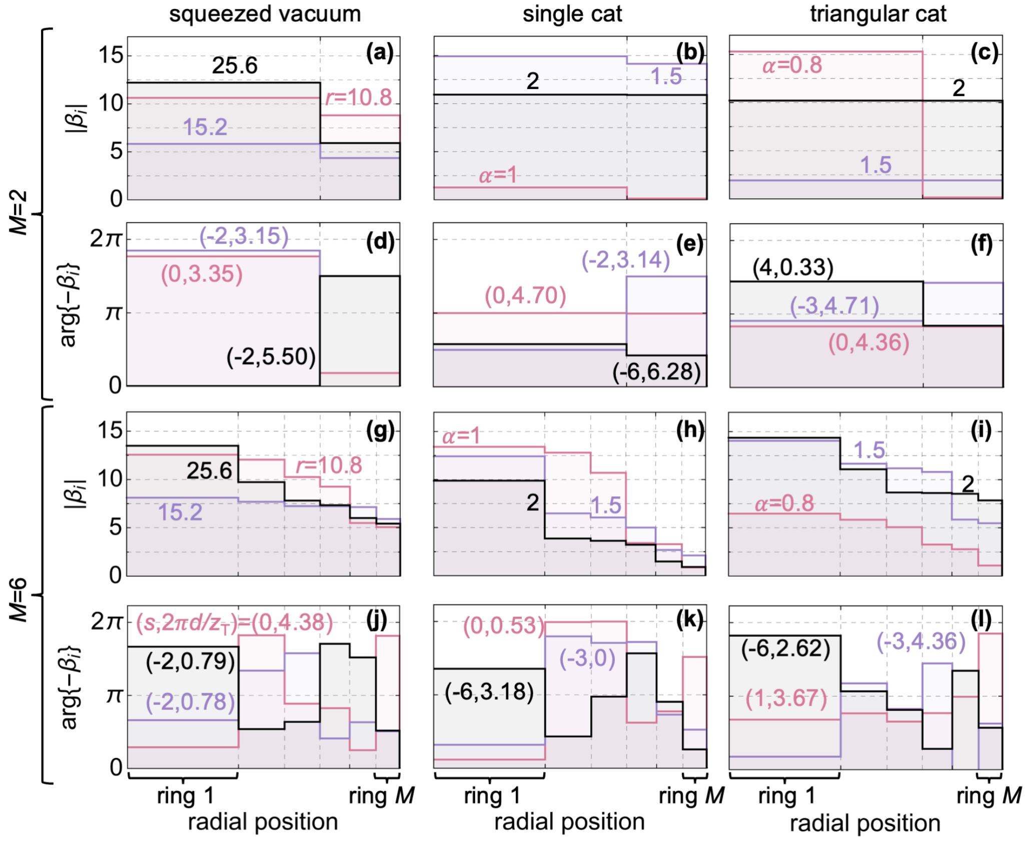

FIG. S2: **IELS coupling strength profiles**. **(a-l)** Optimal coupling coefficients β_i, post-filtering sidebands s, and quadratic phases $2\pi d/z_{\rm T}$ for the target light states marked with black dots in Fig. (5b-d) of the main text. The results of the two-sector ($M=2$) strategy are shown in panels **(a-f)** while the ones corresponding to the six-sector strategy ($M=6$) in **(g-l)**.

B. Natural synthesis of cat states after a single unstructured inelastic electron-light scattering (IELS) interaction

When an electron traverses an electric field distribution $\vec{\mathcal{E}}(\mathbf{r},t)=\vec{\mathcal{E}}(\mathbf{r})\mathrm{e}^{-\mathrm{i}\omega_{\rm L}t}+\mathrm{c.c.}$, arising from the scattering of a laser pulse of photon energy $\hbar\omega_{\rm L}$ onto a nanostructure, its initial wave function $\psi_0(z,t)$ undergoes inelastic electron-light scattering (IELS) modifying its spatial and energetic structure. For relativistic electrons, the exact exit state after traveling a distance d comparable with the Talbot distance $z_{\rm T}=4\pi m_{\rm e}v^3\gamma^3/\hbar\omega_{\rm L}$, within the electron conditions considered in this work, can be found in several works [4, 8, 9] and reads

$$\psi_{\rm IELS}(z,t)=\psi_0(z,t)\sum_{\ell=-\infty}^{\infty}J_\ell(2|\beta|)\,\mathrm{e}^{\mathrm{i}\ell\omega_0(z-vt)/v+\mathrm{i}\ell\arg\{-\beta\}-2\pi\mathrm{i}\ell^2d/z_{\rm T}}, \tag{S19}$$

where $J_\ell(x)$ is the ℓ-th other Bessel function, $\beta=(e/\hbar\omega_{\rm L})\int_{-\infty}^{\infty}dz\,\mathcal{E}_z(\mathbf{R},z)\,\mathrm{e}^{-\mathrm{i}\omega_{\rm L}z/v}$. By comparing the electron wave function used to obtain Eq. (S10a) and Eq. (S19), we identify the coefficients c_n typical of a IELS interaction, which therefore read $c_n=J_n(2|\beta|)\,\mathrm{e}^{\mathrm{i}n\arg\{-\beta\}}$. Now, we want to study the effect of the $n_{\rm max}$ IELS electron coefficients $c_s,\dots,c_{s+n_{\rm max}}$ on the generated light state. In order to do that, we assume them to vanish for $n>s+n_{\rm max}$, namely we take

$$c_n=\begin{cases}J_n(2|\beta|)\,\mathrm{e}^{\mathrm{i}n\arg\{-\beta\}}/\big[\sum_{m=s}^{s+n_{\rm max}}J_m^2(2|\beta|)\big]^{1/2}, & \text{for } s\le n\le s+n_{\rm max},\\ 0, & \text{otherwise,}\end{cases} \tag{S20}$$

where the denominator plays the role of a normalization constant.

For a very high electron-light coupling, we can take the asymptotic expansion $J_n(2|\beta|)\approx(\pi|\beta|)^{-1/2}\cos[2|\beta|-n\pi/2-\pi/4]$ [10] valid for filtering values $(n_{\rm max}+s)^2\ll 2|\beta|$. By plugging the previous expression into Eq. (S20) with $k=0$ and working out the normalization factor P_F with the help of the relation $\sum_{k=0}^{n}\lambda^k/k!=$

$\mathrm{e}^{\lambda}\,\Gamma(n+1,\lambda)/n!$, where $\Gamma(n,x)=\int_x^\infty t^{n-1}\mathrm{e}^{-t}dt$ is the incomplete gamma function, we obtain

$$\alpha_{p,n}=\begin{cases}\langle n|\chi\rangle\left[1+\mathrm{e}^{\mathrm{i}(s\pi+\pi/2-4|\beta|)}(-1)^n\right]/P_F^{1/2}, & \text{for } 0\le n\le n_{\max},\\ 0, & \text{otherwise},\end{cases}\tag{S21}$$

where $\chi=-\mathrm{i}\beta_0\mathrm{e}^{\mathrm{i}\arg\{-\beta\}}$ the dividing factor can now be written in the compact form $P_F=2\big[\Gamma(n_{\max}+1,\beta_0^2)+(-1)^s\mathrm{e}^{-2\beta_0^2}\sin(4|\beta|)\Gamma(n_{\max}+1,-\beta_0^2)\big]/n_{\max}!$. Eq. (S21) needs to be compared with the photon-number coefficients of a cat state $\langle n|\mathrm{cat}_\theta^\alpha\rangle=\langle n|\alpha\rangle[1+\mathrm{e}^{\mathrm{i}\theta}(-1)^n]/[2+2\cos(\theta)\mathrm{e}^{-2|\alpha|^2}]^{1/2}$ to realize that a cat state with $\theta=s\pi+\pi/2-4|\beta|$ and $\alpha=\chi$ is created by a single electron-light modulation, filtering, and post-filtering with a precision depending on the value of $s+n_{\max}$.

S4. MODULATION OF ENERGY COEFFICIENTS THROUGH LATERALLY-STRUCTURED IELS INTERACTION AND THEIR OPTIMIZATION

A. Energy coefficients in the interaction region

In this section, we report a variation of the method presented in Ref. [11] to produce approximated electron energy coefficients c_ℓ as close as possible to the ones needed to crate a given target light state $\alpha_n^{\rm targ}$, according to the relation in Eq. (S16). This method leverages a wide electron beam traversing a near-field structured in concentric circular sections [see Fig. 5a in the main text] at plane $z=0$ which is then focused to the focal point $(\mathbf{R},z)=(0,z_0+f)$ by an axially symmetric and aberration-free converging lens placed at z_0, with radius $R_{\max}$ and numerical aperture $\mathrm{NA}\approx R_{\max}/f$.

The time-dependent electron wave after passing through such interaction can be written as the three-dimensional extension of Eq. (S19) [11]

$$\psi_{\rm IELS}(\mathbf{r},t)=\psi_0(\mathbf{r},t)\,\mathrm{e}^{-\mathrm{i}q_0z}\sum_{\ell=-\infty}^{\infty}J_\ell(2|\beta(\mathbf{R})|)\mathrm{e}^{\mathrm{i}q_\ell z+\mathrm{i}\arg\{-\beta(\mathbf{R})\}-\mathrm{i}\ell\omega_{\rm L}t},\tag{S22}$$

where we have introduced the longitudinal momentum $q_\ell\approx q_0+\ell\omega_{\rm L}/v-\ell^2/z_{\rm T}$ corresponding to an energy $E_0^e+\hbar\omega_{\rm L}\ell$. If we assume the electron to be well collimated and covering the entire extension of the interaction zone, we can take $\psi_0(\mathbf{r},t)=\psi_0\,\mathrm{e}^{\mathrm{i}q_0z-\mathrm{i}E_0^et/\hbar}$. The action of the converging lens can be expressed by multiplying energy amplitude of Eq. (S22) with $\theta(R_{\max}–R)\mathrm{e}^{-\mathrm{i}q_\ell R^2/2f}$ which at the lens' plane becomes

$$\psi^{\rm lens}_{\rm IELS}(\mathbf{R},z_0,t)=\psi_0\,\mathrm{e}^{-\mathrm{i}E_0^et}\sum_{\ell=-\infty}^{\infty}J_\ell[2|\beta(\mathbf{R})|]\,\mathrm{e}^{\mathrm{i}q_\ell z_0+\mathrm{i}\arg\{-\beta(\mathbf{R})\}-\mathrm{i}\ell\omega_{\rm L}t}\mathrm{e}^{-\mathrm{i}q_\ell R^2/2f}.\tag{S23}$$

Now, we use scalar diffraction theory [12, 13] to propagate the wave function of Eq. (S23) from the plane z_0 to the focal plane z_0+f. Indeed, from the knowledge of an electron wave $\psi_\ell(\mathbf{R},z_0)$ with total momentum q_ℓ at z_0, $\psi_\ell(\mathbf{R},z_s)$ can be obtained through the expression

$$\begin{aligned}\psi_\ell(\mathbf{R},z_s)&=\frac{1}{(2\pi)^2}\int d^2\mathbf{Q}\,\mathrm{e}^{\mathrm{i}\mathbf{Q}\cdot\mathbf{R}+\mathrm{i}q_z^\ell(z_s-z_0)}\int d^2\mathbf{R}'\psi_\ell(\mathbf{R}',z_0)\,\mathrm{e}^{-\mathrm{i}\mathbf{Q}\cdot\mathbf{R}'}\\&\approx\frac{-\mathrm{i}q_\ell}{2\pi(z_s-z_0)}\int d^2\mathbf{R}'\psi_\ell(\mathbf{R}',z_0)\,\mathrm{e}^{\mathrm{i}|\mathbf{R}-\mathbf{R}'|^2q_\ell/2(z_s-z_0)+\mathrm{i}q_\ell(z_s-z_0)},\end{aligned}\tag{S24}$$

where the last line was obtained by taking the paraxial approximation $q_z^\ell=\sqrt{q_\ell-Q^2}\approx q_\ell-Q^2/2q_\ell$ and the integral $\int_0^\infty dx\,x\,\mathrm{e}^{-\mathrm{i}ax^2}J_0(bx)=(-\mathrm{i}/2a)\mathrm{e}^{\mathrm{i}b^2/4a}$ [Eq. 6.631-4 of Ref. 14]. By applying Eq. (S24) to each energy component of Eq. (S23) and by employing the axial symmetry of the field, that implies $\beta(\mathbf{R})\equiv\beta(R)$, one arrives to the expression

$$\psi^{\rm lens}_{\rm IELS}(\mathbf{R},z_s,t)=\frac{-\mathrm{i}\psi_0f^2}{(z_s-z_0)}\mathrm{e}^{-\mathrm{i}E_0^et/\hbar}\sum_{\ell=-\infty}^{\infty}b_\ell(\mathbf{R},z_s-z_0)\,\mathrm{e}^{\mathrm{i}q_\ell z_s-\mathrm{i}\ell\omega_{\rm L}t},\tag{S25a}$$

$$b_\ell(\mathbf{R},z_s-z_0)=q_\ell\,\mathrm{e}^{\mathrm{i}R^2q_\ell/2(z_s-z_0)}\int_0^{\rm NA}d\theta\,\theta\,J_0\left(\frac{Rfq_\ell\theta}{z_s-z_0}\right)J_\ell[2|\beta(\theta)|]\,\mathrm{e}^{-\mathrm{i}\theta^2q_\ell f(z_s-z_0-f)/2(z_s-z_0)}\mathrm{e}^{\mathrm{i}\ell\arg\{-\beta(\theta)\}}.\tag{S25b}$$

Since we are interested in the electron wave function close to interaction with the cavity, assumed to be placed at the focus, and since the coefficients $c_\ell(\mathbf{R},z_s-z_0)$ do not vary considerably along its extension $\sim100\ \mu$m for

electron kinetic energies ~ 100 keV, NA $\sim 2\times10^{-4}$, we take $b_\ell(\mathbf{R},z_s-z_0)\approx b_\ell(\mathbf{R}=0,f)$ in Eq. (S25a). In addition, by approximating q_ℓ with its second order Taylor expansion in the exponential of Eq. (S25a) and with q_0 in Eq. (S25b), we transform the former equation at $z_s=z_0+f+z$ into

$$\psi^{\rm lens}_{\rm IELS}(\mathbf{R},z_0+f+z,t)\approx -{\rm i}\psi_0 f q_0 {\rm e}^{-{\rm i}E^e_0 t/\hbar+{\rm i}q_0(z_0+f+z)}\sum_{\ell=-\infty}^{\infty} c_\ell\, {\rm e}^{{\rm i}\ell\omega_{\rm L}(z_0+f+z)/v-{\rm i}\ell\omega_{\rm L}t}, \tag{S26}$$

where now $c_\ell={\rm e}^{-2\pi{\rm i}\ell^2(z_0+f)/z_{\rm T}}\int_0^{\rm NA} d\theta\theta J_\ell[2|\beta(\theta)|]\,{\rm e}^{{\rm i}\ell{\rm arg}\{-\beta(\theta)\}}$. In the configuration sketched in Fig. (5a) of the main text, $\beta(\theta)$ is assumed to take constant value β_i in the i-th of the M concentric sectors of equal normalized area a. This directly leads to the simple form $c_\ell=(a/\pi){\rm e}^{-2\pi{\rm i}\ell^2 d/z_{\rm T}}\sum_{i=1}^M J_\ell(2|\beta_i|)\,{\rm e}^{{\rm i}\ell{\rm arg}\{-\beta_i\}}$ with $d=z_0+f$ used to maximize the fidelity of the light state generated by Eq. (S16) and a target state. Because of the normalization condition, the prefactor in c_ℓ does not play any role in the optimization process and thus its output is independent of a. Finally, in order to match the form of the electron state in Eq. (S26) with the one used to arrive at Eq. (S10a), we absorb the phase $\omega_{\rm L}(z_0+f)/v$ into ${\rm arg}\{-\beta_i\}$.

B. Optimization method

To find the optimal electron states capable of synthesizing the quantum light states analyzed in this work, the IELS coefficients β_i and the propagation distance d are found by employing a random search algorithm combined with a steepest descent method. A maximum number of iterations of 2000 for the steepest descent together with 3000 random initial conditions ensured convergence of the results. In Fig. (S2), we report the optimal coefficients of specific instances shown in Fig. (5b-d) of the main text.

[1] V. Di Giulio, O. Kfir, C. Ropers, and F. J. García de Abajo, ACS Nano **15**, 7290 (2021).
[2] F. J. García de Abajo, Rev. Mod. Phys. **82**, 209 (2010).
[3] R. Remez, A. Karnieli, S. Trajtenberg-Mills, N. Shapira, I. Kaminer, Y. Lereah, and A. Arie, Phys. Rev. Lett. **123**, 060401 (2019).
[4] F. J. García de Abajo and V. Di Giulio, ACS Photonics **8**, 945 (2021).
[5] Y. Pan and A. Gover, J. Phys. Commun. **2**, 115026 (2018).
[6] O. Kfir, V. Di Giulio, F. J. García de Abajo, and C. Ropers, Sci. Adv. **7**, eabf6380 (2021).
[7] K. Tsuno and D. Ioanoviciu, *Chapter Six - Application of Wien Filters to Electrons*, vol. 176 (Elsevier, Amsterdam, 2013).
[8] S. T. Park, M. Lin, and A. H. Zewail, New J. Phys. **12**, 123028 (2010).
[9] V. Di Giulio and F. J. García de Abajo, Optica **7**, 1820 (2020).
[10] M. Abramowitz and I. A. Stegun, *Handbook of Mathematical Functions* (Dover, New York, 1972).
[11] F. J. García de Abajo and C. Ropers, Phys. Rev. Lett. **130**, 246901 (2023).
[12] C. J. Bouwkamp, Rep. Prog. Phys. **17**, 35 (1954).
[13] L. Mandel and E. Wolf, *Optical Coherence and Quantum Optics* (Cambridge University Press, Cambridge, 1995).
[14] I. S. Gradshteyn and I. M. Ryzhik, *Table of Integrals, Series, and Products* (Academic Press, London, 2007).
